# Elexacaftor is a CFTR potentiator and acts synergistically with ivacaftor during acute and chronic treatment

**DOI:** 10.1038/s41598-021-99184-1

**Published:** 2021-10-06

**Authors:** Ciaran A. Shaughnessy, Pamela L. Zeitlin, Preston E. Bratcher

**Affiliations:** 1 Department of Pediatrics, National Jewish Health, Denver, CO USA; 2 Department of Pediatrics, University of Colorado Anschutz Medical Center, Aurora, CO USA

**Keywords:** Translational research, Molecular medicine

## Abstract

Cystic fibrosis (CF) is caused by mutations in the cystic fibrosis transmembrane conductance regulator (CFTR), which lead to early death due to progressive lung disease. The development of small-molecule modulators that directly interact with CFTR to aid in protein folding (“correctors”) and/or increase channel function (“potentiators”) have proven to be highly effective in the therapeutic treatment of CF. Notably, incorporation of the next-generation CFTR corrector, elexacaftor, into a triple combination therapeutic (marketed as Trikafta) has shown tremendous clinical promise in treating CF caused by F508del-CFTR. Here, we report on a newly-described role of elexacaftor as a CFTR potentiator. We explore the acute and chronic actions, pharmacology, and efficacy of elexacaftor as a CFTR potentiator in restoring function to multiple classes of CFTR mutations. We demonstrate that the potentiating action of elexacaftor exhibits multiplicative synergy with the established CFTR potentiator ivacaftor in rescuing multiple CFTR class defects, indicating that a new combination therapeutic of ivacaftor and elexacaftor could have broad impact on CF therapies.

## Introduction

Cystic fibrosis (CF) is the most common life-threatening autosomal recessive genetic disease among Caucasians worldwide, and is caused by mutations in a single gene encoding an epithelial chloride channel, the phosphorylation-activated cystic fibrosis transmembrane conductance regulator (CFTR), that result in decreased channel expression and/or function^1–3^. Epithelial chloride transport via CFTR facilitates the osmotic movement of water and is critical in maintaining fluid and electrolyte homeostasis of various epithelial surfaces, notably airway epithelia. Dysfunctional CFTR leads to the over-accumulation of dehydrated mucus in the lung, which impairs the ability of the lung to expel inhaled pathogens, causing chronic infection, inflammation, and fibrosis, and leading to early death due to progressive lung disease.

The development of small-molecule modulators that directly interact with CFTR to increase channel expression and/or function have proven to be highly effective in the therapeutic treatment of CF^4^. Pathogenic mutations in CFTR have been categorized into six distinct functional classes^5^. Class I defects do not produce CFTR protein, Classes II-IV produce functionally defective CFTR protein (II, misfolding/no trafficking; III, impaired gating; IV, decreased conductance), and Classes V-VI produce less or unstable CFTR protein. Individual CFTR modulators have been developed to rectify specific functional defects in Class II-IV mutations. CFTR “correctors” stabilize misfolded protein and increase membrane expression, and CFTR “potentiators” restore channel activity. Clinically, these compounds are employed in a highly precise, mutation-specific manner^6^.

The most common CF-causing mutation of CFTR, F508del, is subject to protein misfolding during assembly. Premature degradation of misfolded F508del-CFTR results in the loss of trafficking of CFTR to the cell surface. Recently, the discovery of the next-generation CFTR modulator, elexacaftor (VX-445) has ushered in the development of a new a triple combination therapeutic marketed as Trikafta in the U.S.A.^7^, which combines the CFTR modulators tezacaftor (VX-661), ivacaftor (VX-770), and VX-445, with impressive clinical results^8–16^. Individually, VX-661 is a CFTR corrector, which stabilizes misfolded F508del-CFTR and increases its trafficking through the Golgi complex to the plasma membrane^17,18^. VX-770 is a CFTR potentiator that rectifies the channel gating defect in F508del-CFTR and other CFTR mutations^19–22^. Marketed as Kalydeco^23^, VX-770 alone has been an important clinical tool in treating many CF-causing mutants, notably G551D and R117H, which exhibit defects in gating and conductance, respectively. Together, VX-661 and VX-770 (marketed as Symdeko^24^) have been widely used in the treatment of CF caused by F508del-CFTR^25–29^. Given the substantially greater efficacy of Trikafta compared to Symdeko in treating CF^15^, there is great interest in more fully understanding the mechanism(s) of action of VX-445 in restoring function to mutant CFTR.

Only a few studies have examined the mechanisms of action of VX-445. In human airway epithelial cell cultures derived from individuals with CF, treatment with VX-445 was found to increase the amount of mature, cell-surface F508del-CFTR compared to VX-661^15^, indicating that VX-445 is a CFTR corrector. When used in combination, VX-445 and VX-661 exhibit multiplicative synergy^15,30^, indicating that these two CFTR correctors have distinct mechanisms of action in stabilizing F508del-CFTR. It has been shown that the stabilizing action of VX-445 occurs through its interaction with the nucleotide binding domain 1 (NBD1) of CFTR^30^.

Only very recently has it been shown that VX-445 can also increase ion conductance through CFTR as a potentiator^31,32^. This potentiating action of VX-445 was first described by Laselva et al. in Class II mutations of CFTR^31^. Very soon after, Veit et al. described the acute potentiation of Class III CFTR mutations by VX-445 and the apparent synergy of VX-445 and VX-770 in co-potentiating Class III CFTR mutations. The recent pursuit of this potentiating action of VX-445 by Laselva et al., Veit et al., and our laboratory underscores the importance of this recent finding. Still, these previous reports left many questions unanswered regarding the potentiating action of VX-445, such as whether channel potentiation by VX-445 or the combination of VX-445 and VX-770 is effective in the chronic term, or whether VX-445 can potentiate Class IV CFTR mutations. Thus the current study provides important additional characterization of the mechanism of VX-445 potentiation of CFTR.

The U.S. Food and Drug Administration is now expanding the drug labels for CFTR modulators based the results of in vitro studies with cell culture systems^7,23,24,33,34^. Thus, in vitro studies describing mutation-specific CFTR modulators and modulator combinations can have tremendous clinical importance. Here, we describe the pharmacology and efficacy of VX-445 as a CFTR potentiator, a newly described mechanism of action for this life-changing drug. Using primary-derived human airway epithelia as well as model cells recombinantly expressing CFTR, we demonstrate that VX-445 exhibits multiplicative synergy with VX-770 in potentiating Class III and IV CFTR mutations (represented by G551D and R117H mutations, respectively), indicating these two compounds have distinct mechanisms of action in potentiating CFTR. Given the versatility of VX-770 in treating multiple CFTR class defects^35,36^ as well as non-CF lung diseases resulting from acquired CFTR dysfunction^37–39^, we expect that the additional classification of VX-445 as a CFTR potentiator will have broad impact in the treatment of CF and non-CF lung disease.

## Results

### Acute potentiation of normal and F508del CFTR by VX-445

**Figure 1:**
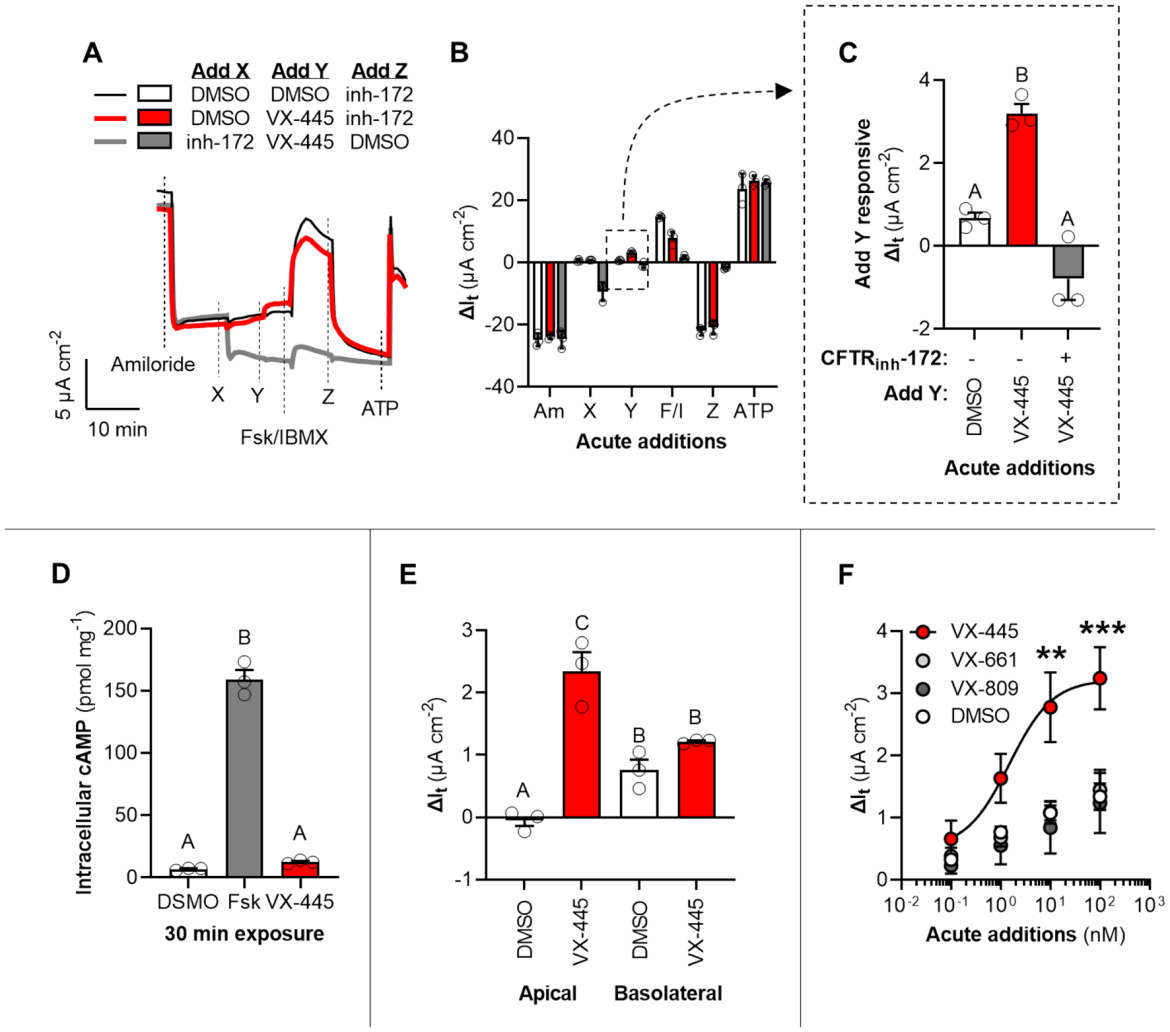
Acute potentiation of CFTR by elexacaftor (VX-445) in primary-derived non-CF human nasal epithelia. (**A**) Representative I_t_ recordings showing response to acute additions of amiloride (100 µM), DMSO, VX-445 (100 nM), Fsk (20 µM)/IBMX (100 µM), and/or CFTR_inh_-172 (100 µM). Acute additions indicated by a letter (X, Y, Z…) correspond to interventions listed in the key in Panel A. (**B**) Changes in I_t_ after the additions of test compounds for the experiment presented in Panel *A*. (**C**) Change in I_t_ after acute addition Y, demonstrating the CFTR-specific potentiation of VX-445. (**D**) Intracellular cAMP after 30 min exposure to DMSO, Fsk, or VX-445. (**E**) Sidedness of immediate (within 5 min) action of VX-445 compared to DMSO controls. (**F**) Concentration–response curve for VX-445 and other CFTR correctors, tezacaftor (VX-661) or lumacaftor (VX-809). Only VX-445 significantly increased I_t_ compared to a DMSO control (denoted by asterisks; EC_50_: 1.50 ± 1.40 nM). See Supplementary Materials for additional experimental details and for supporting data. All data are presented as mean ± standard error. Bars with different letters (A, B, C…) are significantly different from each other (ANOVA; *P* < 0.05). Asterisks indicate specific *P* values: **P < 0.01; ***P < 0.001.

Although VX-445 has been introduced as a CFTR corrector when examined over chronic exposure periods (≥ 24 h)^15^, we observed a detectable acute action of VX-445 in stimulating transepithelial current (I_t_) akin to the action of a CFTR potentiator (Fig. S1). In primary-derived non-CF human nasal epithelial (HNE) cells, acute application of VX-445 resulted in a ~ 3 µA cm^−2^ increase in I_t_, which was approximately one-seventh of the total CFTR-mediated I_t_ detected (~ 20 µA cm^−2^) (Fig. 1A–C). When CFTR was first inhibited with the specific channel inhibitor CFTRinh-172, the acute action of VX-445 in stimulating I_t_ was abolished (Fig. 1C). Acute application of VX-445 had no effect on CFTRinh-172 inhibited I_t_ (indicative of total CFTR-mediated chloride transport) compared to the DMSO control (*P* = 0.996) (Fig. S2A). These results in non-CF HNE demonstrate that VX-445 stimulated I_t_ in a CFTR-dependent manner but did not affect total CFTR-mediated I_t_, indicating that VX-445 may also be acting to potentiate CFTR in addition to its previously established mechanism of action as a CFTR corrector. To investigate the possibility that cAMP/PKA activation and not channel potentiation was the cause of CFTR-mediated I_t_ stimulation by VX-445, we measured intracellular cAMP after 30 min exposure to forskolin (Fsk), VX-445, or a DMSO control. Exposure of airway epithelia to Fsk significantly increased cellular cAMP over tenfold from the DMSO control (*P* < 0.0001) but VX-445 did not alter cellular cAMP (*P* = 0.670) (Fig. 1D).

Characteristically, CFTR potentiators exhibit immediate action when applied to the apical airway surface, the site of CFTR expression^22,40^. We separately applied VX-445 or a DMSO control to the apical and basolateral baths. An immediate action of VX-445 (i.e., significantly greater ΔI_t_ over the DMSO control within 5 min) was only observed after application to the apical surface and not the basolateral surface (Figs. 1E and S2B). To assess the uniqueness of this apparent potentiating action of VX-445 among other established CFTR correctors, we performed a concentration–response analysis with VX-445, VX-661, VX-809 (lumacaftor), and a DMSO control. The aim of this experiment was to establish the EC50 of the acute potentiating action of VX-445 and compare this acute action with other CFTR correctors within this concentration range. Only a titration of VX-445 increased I_t_ above the DMSO control and was better fit by a dose–response curve (three-parameter) than a straight line (*P* = 0.010) (Figs. 1F and S2C). The acute potentiating effect of VX-445 appeared saturating at ~ 100 nM (I_t,max_ = 3.22 ± 0.384 µA cm^−2^) with an EC_50_ of 1.50 ± 1.40 nM (*n* = 12).

**Figure 2:**
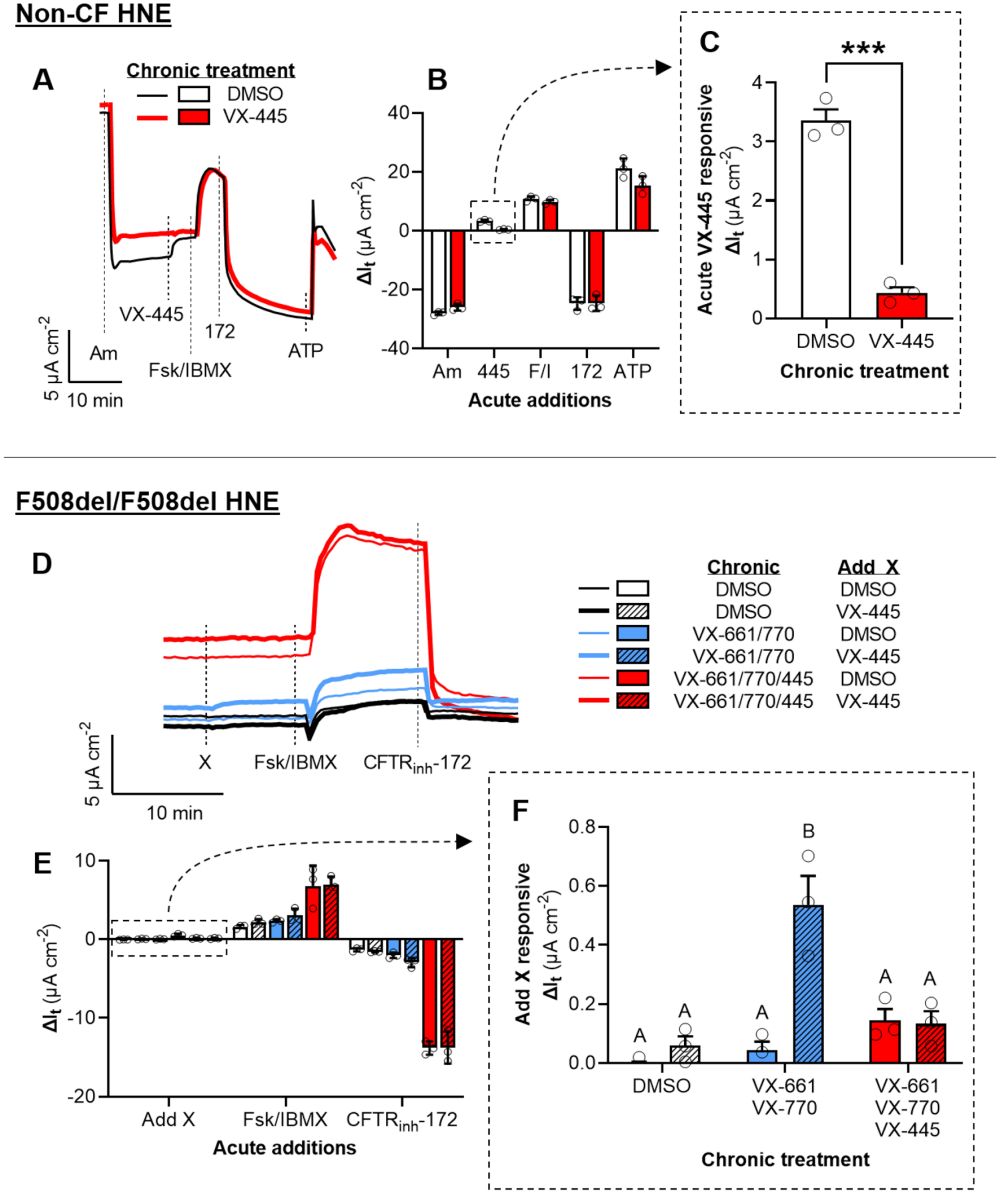
Effect of chronic treatment with elexacaftor (VX-445) includes its actions as both a CFTR corrector and potentiator. (**A**) Representative I_t_ recordings of non-CF HNE treated for 24 h with DMSO or VX-445 (3 µM). (**B–C**) Changes in I_t_ after the additions of test compounds for the experiment presented in Panel **A**. The potentiating action of VX-445 accounts for the differences in post-amiloride I_t_ between DMSO and VX-445 treated cultures. (**D**) Representative I_t_ recordings of F508del-HNE treated for 24 h with tezacaftor (VX-661; 3 µM), ivacaftor (VX-770; 100 nM), and/or VX-445 (3 µM chronic; 100 nM acute). (**E–F**) Changes in I_t_ after the additions of test compounds for the experiment presented in (**D)**. The triple combination of VX-661/77/445 significantly increased CFTR-mediated I_t_. Acute action of VX-445 in stimulating I_t_ was only observed in VX-661/770 treated F508del/F508del nasal cells. See SI for additional experimental details and for supporting data. All data are presented as mean ± standard error. Bars with different letters (A, B, C…) are significantly different from each other (ANOVA; *P* < 0.05). Asterisks indicate specific *P* values: ***P < 0.001.

Next, we sought to better characterize and differentiate the dual roles of VX-445 as a CFTR corrector and potentiator using HNE cultures derived from non-CF individuals and those homozygous for the F508del CFTR mutation (F508del-HNE). We chronically exposed (24 h) non-CF HNE cells to VX-445 or a DMSO control, then assessed the acute potentiating action of VX-445. Acute application of VX-445 increased I_t_ in the DMSO control by ~ 3 µA cm^−2^ but had no effect in epithelia exposed chronically to VX-445 (*P* = 0.0002) (Fig. 2A–C). After subsequent additions of Fsk/IBMX and CFTRinh-172, we observed that chronic treatment with VX-445 resulted in an increase in constitutive CFTR activity (Fig. S3B; calculated as the difference in I_t_ from values obtained after amiloride exposure to values obtained after CFTRinh-172 exposure^41^) but no change in maximal CFTR-mediated I_t_ (calculated as the CFTR_inh_-172 responsive ΔI_t_) compared to the DMSO control (*P* > 0.999) (Figs. 2B and S3A).

We chronically exposed F508del-HNE cells to one of three treatments: (i) a DMSO control, (ii) the double combination VX-661/770 (i.e., Symdeko), or the triple combination VX-661/770/445 (i.e., Trikafta). Then, we acutely exposed the epithelia to either VX-445 or a DMSO control during electrophysiological analysis. As expected, F508del-HNE cells treated with the triple combination exhibited much greater total CFTR-mediated I_t_ compared to either the double combination or the DMSO control (*P* < 0.0001; *n* = 6) (Figs. 2D–E and S3C–D). The dramatic difference between F508del-HNE cells chronically treated with and without VX-445 is evidence of the already established role for VX-445 as a highly effective CFTR corrector^15^. Acute application of VX-445 stimulated I_t_ above the DMSO control only in the F508del-HNE cells treated with the double combination of VX-661/770 (*P* = 0.0002) (Fig. 2F).

We also found similar acute potentiating and chronic correcting actions of VX-445 in the widely-used model cell line in CF research, Fischer rat thyroid (FRT) cells recombinantly expressing normal CFTR (WT-FRT) or F508del-CFTR (F508del-FRT) (Fig. S4A–F).

### Synergism of VX-770 and VX-445 in restoring channel function to G551D-CFTR

**Figure 3:**
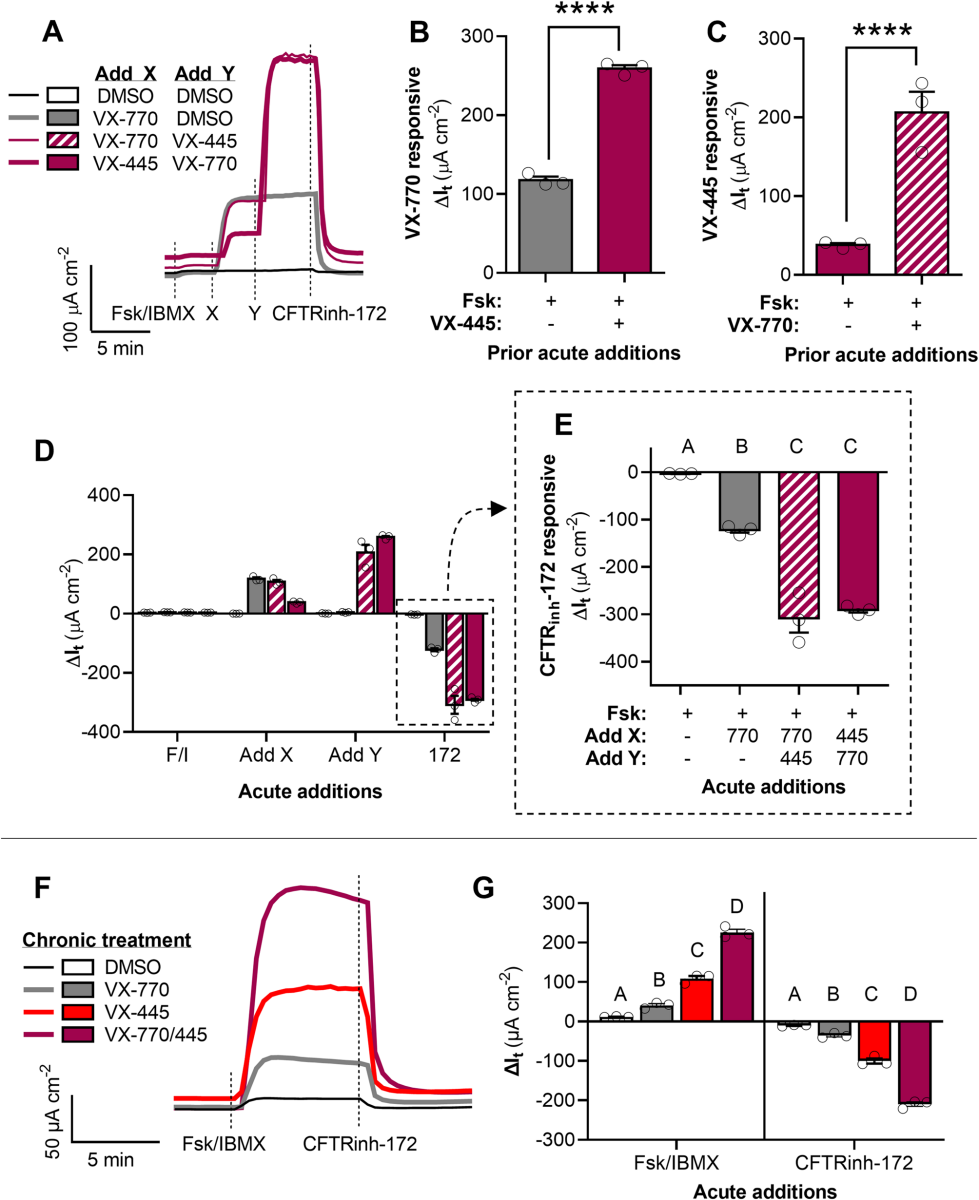
Synergism of ivacaftor (VX-770) and elexacaftor (VX-445) in potentiating G551D-CFTR in FRT cells. (**A**) Representative I_t_ recordings of FRT cells expressing human G551D-CFTR showing acute actions of VX-770 and VX-445. (**B–C**) Changes in I_t_ after acute addition of VX-770 in the absence and presence of VX-445 (**B**) and in response to the acute addition of VX-445 in the absence and presence of VX-770 (**C**). (**D–E**) Changes in I_t_ after the additions of test compounds for the experiment presented in (**A)**. G551D-CFTR mediated I_t_ is greatest after acute potentiation by both VX-770 and VX-445. (**F**) Representative I_t_ recordings of FRT cells expressing human G551D-CFTR treated for 24 h with DMSO, VX-770, and/or VX-445. (**G**) Changes in I_t_ after the additions of test compounds for the experiment presented in (**F)**. G551D-CFTR mediated I_t_ is greatest after chronic treatment by both VX-770 and VX-445. See SI for additional experimental details and for supporting data. All data are presented as mean ± standard error. Bars with different letters (A, B, C…) are significantly different from each other (ANOVA; *P* < 0.05). Asterisks indicate specific *P* values: ****P < 0.0001.

With this role for VX-445 as a CFTR potentiator having been recently discovered, we sought to examine its efficacy in restoring channel function to G551D-CFTR, which characteristically exhibits defective channel gating. In FRT cells recombinantly expressing G551D-CFTR (G551D-FRT), we compared the actions of VX-770 and VX-445 on increasing channel function. After activation by Fsk/IBMX, acute application of VX-445 significantly increased G551D-CFTR mediated I_t_ by 38 ± 2 µA cm^−2^ and acute application of VX-770 significantly increased G551D-CFTR mediated I_t_ by 118 ± 4 µA cm^−2^, a significant difference (*P* < 0.0001) (Fig. 3A–E and S5). Acute application of both VX-770 and VX-445, resulted in a net ΔI_t_ of 232 ± 17 µA cm^−2^, significantly greater than that after application of VX-770 alone (*P* = 0.002), VX-445 alone (*P* < 0.0001), or the sum of their individual effects on I_t_ (~ 156 µA cm^−2^), indicating that the actions of VX-770 and VX-445 as CFTR potentiators are multiplicatively synergistic. Specifically, the effectiveness VX-770 nearly doubled when applied in the presence of VX-445, and effectiveness of VX-445 was over fivefold greater when applied in the presence of VX-770 (Fig. 3B–C).

Chronic treatment of G551D-FRT cells with the combination VX-770/445 increased G551D-CFTR mediated I_t_ (Fig. 3F–G). Compared to a DMSO control (− 10.1 ± 1.1 µA cm^−2^), chronic treatment with VX-770 increased CFTR_inh_-172 inhibited ΔI_t_ by over threefold (− 35.8 ± 3.4 µA cm^−2^; *P* = 0.021). Chronic treatment with VX-445 resulted in even greater CFTR_inh_-172 inhibited ΔI_t_ (− 100.4 ± 6.6 µA cm^−2^), significantly more than treatment with VX-770 alone (*P* < 0.0001). Interestingly, the larger effect of chronic treatment with VX-445 compared to chronic treatment with VX-770 contrasts with the acute effects of these potentiators, in which acute application of VX-445 produces a smaller ΔIt than acute application of VX-770. Chronic treatment with the combination VX-770/445 resulted in the greatest CFTR_inh_-172 inhibited ΔI_t_ (− 209.7 ± 5.8 µA cm^−2^), significantly more than either VX-770 or VX-445 alone (*P* < 0.0001), which mirrors what was observed on an acute timeline.

We also found that VX-445 potentiates R117H-CFTR expressed in FRT cells (Fig. S6A–B). Like in G551D-FRT cells, the combination of VX-445 and VX-770 resulted in the greatest R117H-CFTR mediated I_t_. Unlike in G551D-FRT cells, VX-445 and VX-770 did not exhibit the same high degree of synergy in potentiating R117H-CFTR (Fig. S6C-D).

**Figure 4:**
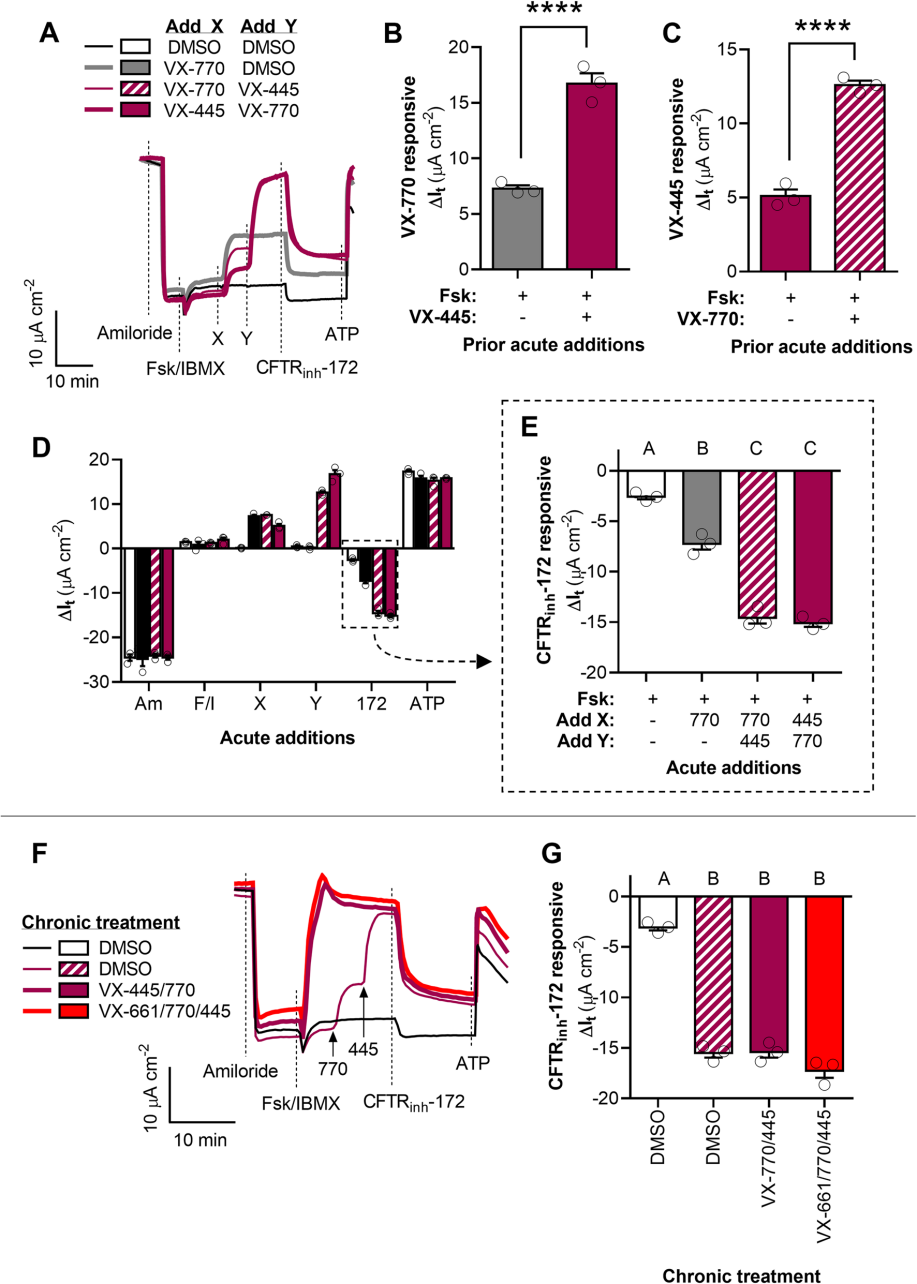
Synergism of ivacaftor (VX-770) and elexacaftor (VX-445) in potentiating G551D-CFTR in HNE cells. (**A**) Representative I_t_ recordings of G551D-HNE cells showing acute actions of VX-770 and VX-445. (**B–C**) Changes in I_t_ after acute addition of VX-770 in the absence and presence of VX-445 (**B**) and in response to the acute addition of VX-445 in the absence and presence of VX-770 (**C**). (**D–E**) Changes in I_t_ after the additions of test compounds for the experiment presented in (**A)**. G551D-CFTR mediated I_t_ is greatest after acute potentiation by both VX-770 and VX-445. (**F**) Representative I_t_ recordings of G551D-HNE treated for 24 h with DMSO, the double combination of VX-770 and VX-445, or the triple combination of VX-661, VX-770, and VX-445 (i.e., Trikafta). (**G**) CFTR_inh_-172 inhibited I_t_ for the experiment presented in (**F)**. See SI for additional experimental details and for supporting data. All data are presented as mean ± standard error. Bars with different letters (A, B, C…) are significantly different from each other (ANOVA; *P* < 0.05). Asterisks indicate specific *P* values: ****P < 0.0001.

Our results in HNE cells derived from an individual homozygous for the G551D mutation (G551D-HNE cells) mirrored what we observed in G551D-FRT cells. After activation by Fsk/IBMX, acute application of VX-445 significantly increased G551D-CFTR mediated I_t_ by 5.1 ± 0.4 µA cm^−2^ and acute application of VX-770 significantly increased G551D-CFTR mediated I_t_ by 7.6 ± 0.1 µA cm^−2^, a significant difference (*P* < 0.0001) (Fig. 4A–E and S7A). Acute application of both VX-770 and VX-445, resulted in a net ΔI_t_ of 15.1 ± 0.3 µA cm^−2^, significantly greater than that after application of VX-770 alone (*P* = 0.0001), VX-445 alone (*P* < 0.0001), or the sum of their individual effects on I_t_ (~ 12.5 µA cm^−2^), replicating the multiplicative synergism as CFTR potentiators we first observed in G551D-FRT cells.

Chronic treatment of G551D-HNE cells with the combination VX-770/445 increased G551D-CFTR mediated I_t_ to the same levels as chronic treatment with the triple combination VX-661/770/445 (~ 12 μA cm^−2^ above the DMSO control), and both were equivalent to the levels achieved by acute treatment with VX-770 and VX-445 (Fig. 4F–G). The levels of G551D-CFTR mediated I_t_ achieved by chronic treatment with VX-445 alone were statistically equivalent to those achieved by treatment with VX-770 alone (*P* = 0.091), but the combination VX-770/445 increased G551D-CFTR mediated I_t_ significantly more than either VX-770 (*P* < 0.0001) or VX-445 (*P* = 0.004) separately (Fig. S8A–B).

## Discussion

We have demonstrated that, in addition to its established role as a CFTR corrector, VX-445 is also an effective potentiator of normal CFTR and the most common pathogenic CFTR variants across mutation Classes II, III, and IV. This work validates and expands upon previous studies on the potentiating action of VX-445 by Laselva et al.^31^ and Veit et al.^32^ by comprehensively characterizing the potentiating action of VX-445 and by examining both the acute and chronic effects of VX-445 potentiation of normal CFTR and CFTR of several different class mutations.

By definition, a potentiator is molecular agent that increases the flow of ions through an activated channel^22^. With respect to CFTR, a potentiator can only improve CFTR-mediated chloride transport if CFTR is expressed on the cell surface and activated by the presence of a cAMP/PKA signaling pathway that phosphorylates CFTR. In the case of Class II CFTR mutations such as F508del-CFTR, the action of a potentiator is reliant on the action of correctors in first chaperoning the mutant protein to the cell surface. For Class III–VI CFTR mutations, treatment with potentiators alone can greatly affect channel function and clinical outcomes of individuals with CF^22,42,43^. The wider role for CFTR potentiators in emerging pharmacological treatments for CF across all classes of CFTR mutations underscores the importance of reconsidering VX-445 as both a corrector and a potentiator, to include this newly-discovered mechanism of action.

Although the recent works by Laselva et al. and Veit et al. have been important in the early discovery of the potentiating action of VX-445, examination of VX-445 action with respect to these defining characteristics of a CFTR potentiator was still lacking. Here, we provide several novel lines of evidence that VX-445 fulfills the definition of a CFTR potentiator. First, we show that acute application of VX-445 increases I_t_ in non-CF HNE and that this action is abolished if CFTR was first inhibited. That this acute, I_t_ stimulating action of VX-445, which occurred in the nanomolar concentration range, was not observed from other CFTR correctors indicates that channel potentiation is an additional mechanism of action that is unique to VX-445 among CFTR correctors. The EC_50_ (~ 1.50 nM) of the acute potentiating action of VX-445 on WT-CFTR observed here was similar to the EC_50_ (~ 1.12 nM) of such action by VX-445 on G551D-CFTR observed by Veit et al.^32^. Second, we show that surface expression of functional CFTR is a prerequisite for VX-445 to acutely stimulate ion conductance. For example, in F508del-HNE cells, VX-445 only affected ion conductance across epithelia that had been pre-treated with the CFTR corrector VX-661. Additionally, the surface to which VX-445 is applied to non-CF epithelia dictates the potency of its immediate (within 5 min) action, indicating that VX-445 is acting on cell-surface CFTR on the apical surface. Lastly, the direct action of VX-445 on CFTR was further supported by our results demonstrating that VX-445 did not affect secondary messenger (cAMP) signaling in non-CF HNE cells.

Having established VX-445 as a CFTR potentiator, the most logical next step was to assess its effect on restoring channel activity to Class III and IV CFTR mutations, which are categorized by channel gating and conductance defects, respectively, and highly responsive to potentiators. Veit et al. have previously demonstrated the acute potentiating action of VX-445 on Class III CFTR mutations (G551D and G1244E)^32^. Yet, whether VX-445 had such potentiating action on normal CFTR or Class IV CFTR mutations and whether VX-445 potentiation of Class III and IV mutations was sustained throughout chronic exposure was still unknown. Thus, it was particularly important to compare potentiation by VX-445 to that by the currently available CFTR potentiator, VX-770 for Class III and IV in both the acute and chronic terms. Acute application of VX-770 increased G551D-CFTR mediated I_t_ more so than did VX-445. Given that the doses of VX-770 and VX-445 are used at acutely saturating concentrations, the significant difference in the magnitude of their effects on I_t_ indicates that the two compounds may have distinct mechanisms of action in potentiating G551D-CFTR.

Interestingly, the potentiating effects of VX-770 and VX-445 appeared to be multiplicatively synergistic, a finding that was recently described by Veit et al.^32^. That is, the increase in G551D-CFTR mediated I_t_ from co-treatment with VX-770 and VX-445 was greater than the sum of their individual effects (Fig. 3). This synergy between VX-770 and VX-445 as CFTR potentiators presented here warrants further investigation in the precise pharmacology and ligand–protein interactions of these two compounds. Whereas it has recently been shown that VX-445 affects F508del-CFTR expression by interacting with the NBD1 domain^31^, whether VX-445 potentiates CFTR by interacting at the same location or a different site altogether should be examined. Our observation that VX-445 potentiates normal CFTR (which was not investigated by Laselva et al. or Veit et al.) indicates that the potentiating action of VX-445 is not due to specific interactions with residues causing the gating or conductance defects in these mutant CFTRs. Further insight into the interaction of VX-770 and VX-445 as channel potentiators will inform attempts to screen compounds for their synergism with currently available CFTR modulators, including additional co-potentiators^44,45^, rather than for their efficacy as standalone CFTR modulators. It is also notable that the synergy of co-potentiation by VX-445 and VX-770 appeared to be much greater for G551D-CFTR than R117H-CFTR, indicating that specific structural properties of mutant CFTR may impact responsiveness to this co-potentiating combination of CFTR modulators. Thus, such co-potentiator screens should investigate efficacy across several class mutations of CFTR.

The cellular background can strongly influence the activities of CFTR modulators, particularly CFTR correctors^46^. Given this, while FRT cells are a useful model to evaluate the acute action of CFTR potentiators, the impacts of chronic exposure to VX-445 on G551D function are probably best measured in our primary-derived, fully differentiated HNE. In G551D-HNE cells, chronic treatment with the combination VX-770/445 reflected what was observed on an acute timeline, that rescue of G551D-CFTR was greatest after treatment with both channel potentiators. Generally, the consistency of our results demonstrating that VX-445 is a CFTR potentiator and exhibits synergism with VX-770 across a range of experimental conditions (e.g., experiments on normal CFTR and Class II–IV mutations of CFTR, experiments on acute and chronic timelines, and experiments using HNE and FRT epithelia) supports the reliability of these results.

In this study, we highlighted the dual roles of VX-445 as a corrector and a potentiator. Whereas an acute timeline (< 30 min) can only reveal the potentiating action of VX-445, its effect on membrane-trafficking of CFTR becomes relevant on a chronic timeline (24 h). That acute application of VX-445 did not stimulate I_t_ in uncorrected F508del-HNE cells but did stimulate I_t_ in VX-661-corrected F508del-HNE cells indicates that the acute stimulation of I_t_ by VX-445 is due to its potentiation of membrane-expressed F508del-CFTR, and not any increase in membrane trafficking of F508del-CFTR. Second, that acute application of VX-445 to F508del-HNE cells treated chronically with VX-661/770 did not increase CFTR-mediated I_t_ to the same levels as those cells treated with the triple combination indicates that the acute action of VX-445 (i.e., as a CFTR potentiator) is not equivalent to the chronic action of VX-445 in rescuing F508del-CFTR function (which includes its actions as a F508del-CFTR corrector and potentiator). This duality of VX-445 functions was not observed in WT- or G551D-HNE cells, which categorically do not require CFTR correctors to rescue channel function. In these cells we showed that the effects of chronic versus acute treatment with the combination of VX-770 and VX-445 were equivalent, indicating that, like VX-770, VX-445 only affects normal CFTR and G551D-CFTR as a channel potentiator. This is also supported by our experiment in G551D-HNE cells demonstrating for the first time that chronic treatment with the combination of VX-770 and VX-445 is just as effective at restoring G551D-CFTR function as chronic treatment with the triple combination (i.e., Trikafta). This result was anticipated as the VX-661 component of Trikafta has been shown to bind to F508del-CFTR and stabilize the protein^30,47^.

Classifying VX-445 as both a CFTR corrector and a channel potentiator has important clinical implications. Consideration of VX-445 as only a CFTR corrector that enhances the trafficking of protein to the cell surface may limit the application of VX-445 to only Class II CFTR mutations, which require pharmacological intervention to improve CFTR membrane trafficking. In light of the present study, there is now evidence to support the use of VX-445 in treating CF in individuals with Class III-VI CFTR mutations. In particular, the double combination of VX-445 alongside VX-770 has a remarkable impact in restoring channel function to G551D- and R117H- CFTR. A limitation of the current study is that our experiments were performed in primary-derived and model epithelia in vitro rather than in vivo. Whether and how precisely our results in vivo translate to the clinical experience in vivo should be discussed. An expanded discussion of the translatability of in vivo studies on CFTR function, particularly on studies examining CFTR modulator efficacies, are discussed further in the Supplementary Text. Recent analyses have shown that epithelial studies on CFTR-mediated ion current in vitro are useful in predicting clinical outcomes such as improved lung function in vivo^48^. It is because of the powerful translatability of CFTR functional studies in vitro that the U.S. Food and Drug Administration has authorized the expansion of CFTR modulator drug labels based on the results of in vitro studies alone^7,23,24,33,34^.

Comparisons of in vitro and in vivo efficacies of VX-770 and the results of the present study indicate that the double combination of VX-770 and VX-445 has tremendous promise as a therapeutic approach. For example, in vitro treatment with VX-770 results in a 3–fourfold increase in G551D-CFTR mediated I_t_ (present study and^22^) over vehicle controls. Clinical trials in individuals harboring at least one G551D-CFTR allele demonstrate that treatment with VX-770 can reduce the difference in lung function from normal level (measured as the percent of predicted forced expiratory volume in 1 s; FEV1) by approximately 30%^43^. Our results demonstrate that the double combination of VX-770 and VX-445 increases G551D-CFTR mediated I_t_ 20-fold over vehicle controls (approximately 3–4 times the efficacy of VX-770 alone). Considering these results in the context of the clinical results of VX-770, clinical benefits of treatment with the double combination of VX-445 and VX-770 seem likely. Based on the results of in vitro studies alone, the drug label for Trikafta has been expanded to include treating CF caused by G551D-CFTR and other Class III–V CFTR mutations, including G551D and R117H^7^. However, here we show that the double combination of VX-770 and VX-445 is equally effective at rescuing G551D-CFTR channel function compared to the triple combination (Fig. 4F–G), indicating that the incorporation of VX-661 in treatment of CF caused by G551D-CFTR mutation may confer no additional benefit. We suspect, based on our results in R117H-FRT cells that this double combination may be similarly effective in treating CF in individuals harboring a wide range of CFTR mutations.

Data from our studies using F508del-, G551D-, and R117H-CFTR demonstrate that the combination VX-770/445 dramatically increases ion conductance through CFTR harboring these mutations. Our results in non-CF HNE demonstrate that VX-770 and VX-445 increase ion conductance even in the absence of exogenous CFTR activating compounds like Fsk/IBMX (present study and^49^). Therefore, the combination VX-770/445 should have the ability to increase CFTR function in all cases where it would be beneficial to do so, including in individuals with CF as well as in individuals who do not have CF but have acquired CFTR dysfunction^38,39,50^. Importantly, any pharmacological means to increase CFTR-mediated ion conductance (such as by the combination VX-770/445) decreases the threshold of surface expression needed to achieve adequate CFTR function. We have demonstrated that constitutive activity of WT-CFTR is increased after chronic treatment with VX-445 (Fig.S3B). Thus, by increasing constitutive CFTR activity, the potency of channel potentiation by the combination VX-770/445 could reduce the required efficacy of emergent read-through agents and cDNA/gene therapies to restore channel function to Class I CFTR mutations that may not ultimately exhibit gating or conductance defects as^3,51^.

In conclusion, through these in vitro studies, we have uncovered a novel mechanism of action for VX-445 as a CFTR potentiator in addition to its previously established mechanism of action as a CFTR corrector. We have shown that when used as a CFTR potentiator, VX-445 has a significant impact in restoring channel function to multiple classes of CFTR mutations. Notably, when used in combination, VX-445 and VX-770 act synergistically to improve ion conductance through mutant CFTR. Given the tremendous clinical importance of VX-770 since its discovery over a decade ago and of VX-445 since its introduction only a few years ago, we suspect the results of the present study will have broad interest to investigators and clinicians studying and treating CF and other forms of lung disease and inspire future clinical investigations.

## Methods

### Cell expansion and maintenance

All specimen collection and experimental methods were carried out in accordance with relevant guidelines and regulations by the National Institutes of Health. Primary human nasal epithelial (HNE) cells were obtained by nasal brushings under informed consent from non-CF individuals and CF individuals homozygous for F508del or G551D CFTR mutations using a protocol approved by the National Jewish Health Institutional Review Board (HS-2832). Nasal brushings were expanded and cultured as previously described^49,52–54^. Briefly, HNE cells were expanded on an irradiated NIH 3T3 feeder layer with Y-27632 Rho-kinase inhibitor, then cultured on collagen-coated, permeable supports at an air–liquid interface (ALI) for 21–28 d to form a differentiated, polarized epithelium. HNE cells were passed 3–4 times before they were used in electrophysiology analyses. Suitable HNE cultures were defined by the visible presence of beating cilia and secreted mucus, adequate transepithelial resistance (R_t_ > 100 Ω cm^−2^), and adequately negative transepithelial potential difference (V_t_ < -5 mV). Fischer rat thyroid (FRT) cells exogenously expressing wild type (WT)-, F508del-, G551D-, or R117H-CFTR were cultured at a liquid–liquid interface for 7–14 d prior to electrophysiological analyses. For further details, see Supplementary Materials and Methods.

### Electrophysiological analyses and drug treatment

Ussing chamber electrophysiological analyses using cultured HNE and FRT cells were conducted under current clamp mode (± 5 mV pulses every 20 ms), which allows V_t_ to vary freely with the continuous monitoring of V_t_, R_t_, and transepithelial current equivalents (I_t_; µA cm^−2^). HNE and FRT cultures were analyzed under symmetrical or gradient (basolateral-to-apical) chloride conditions, respectively. Unless otherwise noted in figure legends, test compounds applied acutely were used at the following concentrations: amiloride (100 μM), VX-770 (1 μM), VX-445 (100 nM) forskolin (20 μM) and 3-isobutyl-1-methylxanthine (100 μM) (Fsk/IBMX or F/I), CFTR_inh_-172 (10 μM), ATP (100 μM). Chronic (24 h) treatment with CFTR modulators were used at the following concentrations: VX-661 (3 μM), VX-770 (100 nM), VX-445 (3 μM). For further details, see Supplementary Materials and Methods.

### Intracellular cAMP determination

Intracellular cAMP was determined using a commercial ELISA kit (ADI-900-163, ENZO Life Sciences) following the manufacture’s potocol. After a 30 min exposure to DMSO, Fsk, or VX-445, HNE cultures on transwell inserts were removed from culture media, blotted dry, and lysed in manufacturer-provided buffer. Once lysed, samples were spun down and the supernatant was used analyzed by ELISA for cAMP concentration alongside cAMP standard curve. Intracellular cAMP levels are expressed as pmol cAMP mg protein^−1^. Protein content of supernatant samples were determined using the Pierce BCA Protein Assay (Thermo Fisher Scientific, Rockford, IL).

### Calculations and statistical analysis

Transepithelial potential difference (*V*_t_) was determined as: *V*_apical_ − *V*_basolateral_. The acute changes in *I*_t_ and *V*_t_ after the acute addition of test compounds (Δ*I*_t_ and Δ*V*_t_, respectively) were calculated as *I*_t(post)_ − *I*_t(pre)_ and *V*_t(post)_ − *V*_t (pre)_, respectively. As indicated in the figure captions, statistical analyses of experiments were made using two-sample *t*-tests, or one-, two-, or three-way ANOVA with Tukey’s post hoc analyses). An α value of 0.05 was selected to denote statistical significance and all data were presented as mean ± standard error, unless otherwise noted. Figure assembly and all statistical analyses were completed using GraphPad Prism 6.0 software (GraphPad Software; La Jolla, CA).

## Supplementary Information

Supplementary Information.
