# Author Correction: Elexacaftor is a CFTR potentiator and acts synergistically with ivacaftor during acute and chronic treatment

**DOI:** 10.1038/s41598-021-00539-5

**Published:** 2021-10-25

**Authors:** Ciaran A. Shaughnessy, Pamela L. Zeitlin, Preston E. Bratcher

**Affiliations:** 1 Department of Pediatrics, National Jewish Health, Denver, CO USA; 2 Department of Pediatrics, University of Colorado Anschutz Medical Center, Aurora, CO USA

Correction to: *Scientific Reports* 10.1038/s41598-021-99184-1, published online 06 October 2021

The original version of this Article contained an error in Figure 3 and 4 where the graph for panel C was omitted. The original Figures 3 and 4 and accompanying legends appear below.

**Figure 3:**
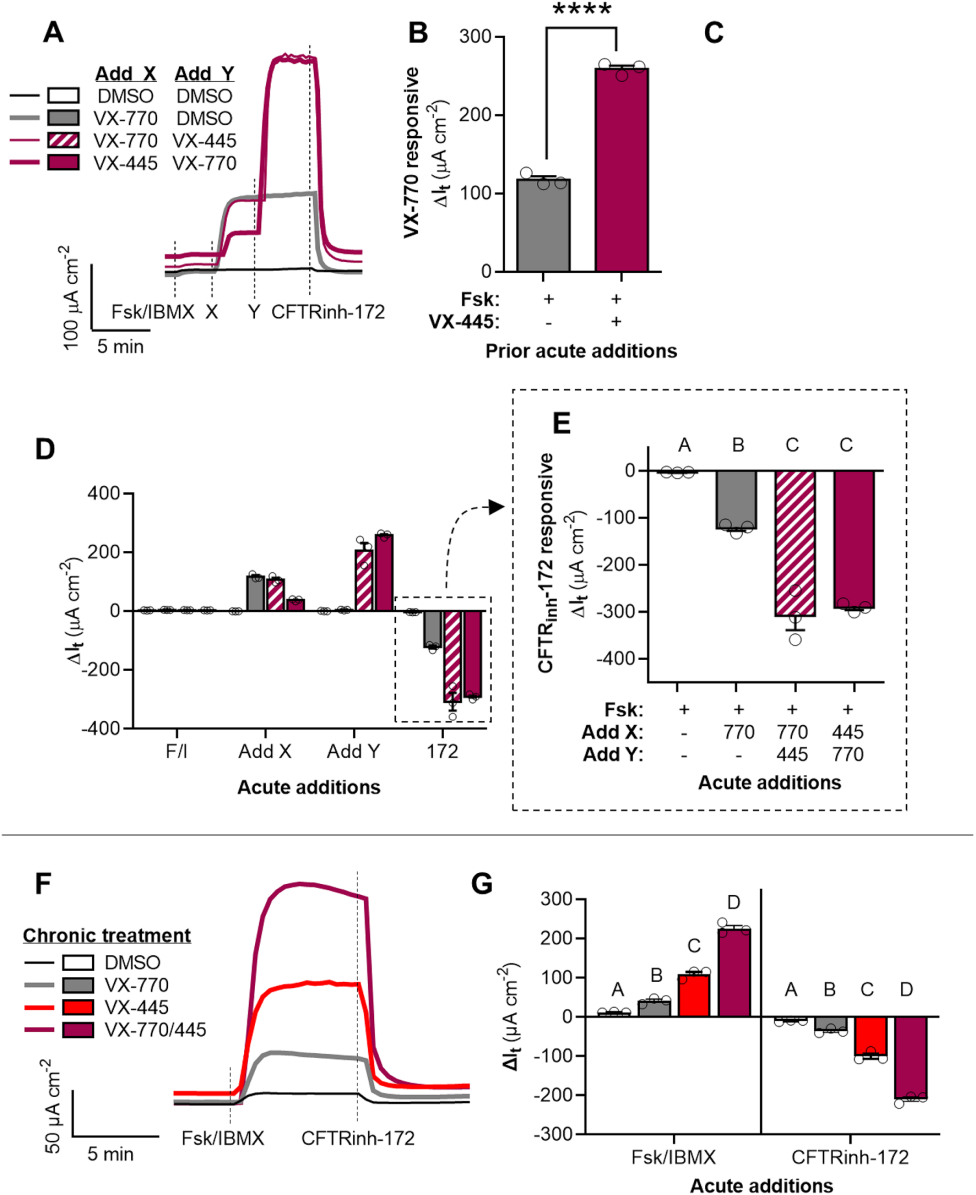
Synergism of ivacaftor (VX-770) and elexacaftor (VX-445) in potentiating G551D-CFTR in FRT cells. (**A**) Representative I_t_ recordings of FRT cells expressing human G551D-CFTR showing acute actions of VX-770 and VX-445. (**B–C**) Changes in I_t_ after acute addition of VX-770 in the absence and presence of VX-445 (**B**) and in response to the acute addition of VX-445 in the absence and presence of VX-770 (**C**). (**D–E**) Changes in I_t_ after the additions of test compounds for the experiment presented in (**A)**. G551D-CFTR mediated I_t_ is greatest after acute potentiation by both VX-770 and VX-445. (**F**) Representative I_t_ recordings of FRT cells expressing human G551D-CFTR treated for 24 h with DMSO, VX-770, and/or VX-445. (**G**) Changes in I_t_ after the additions of test compounds for the experiment presented in (**F)**. G551D-CFTR mediated I_t_ is greatest after chronic treatment by both VX-770 and VX-445. See SI for additional experimental details and for supporting data. All data are presented as mean ± standard error. Bars with different letters (A, B, C…) are significantly different from each other (ANOVA; *P* < 0.05). Asterisks indicate specific *P* values: ****P < 0.0001.

**Figure 4:**
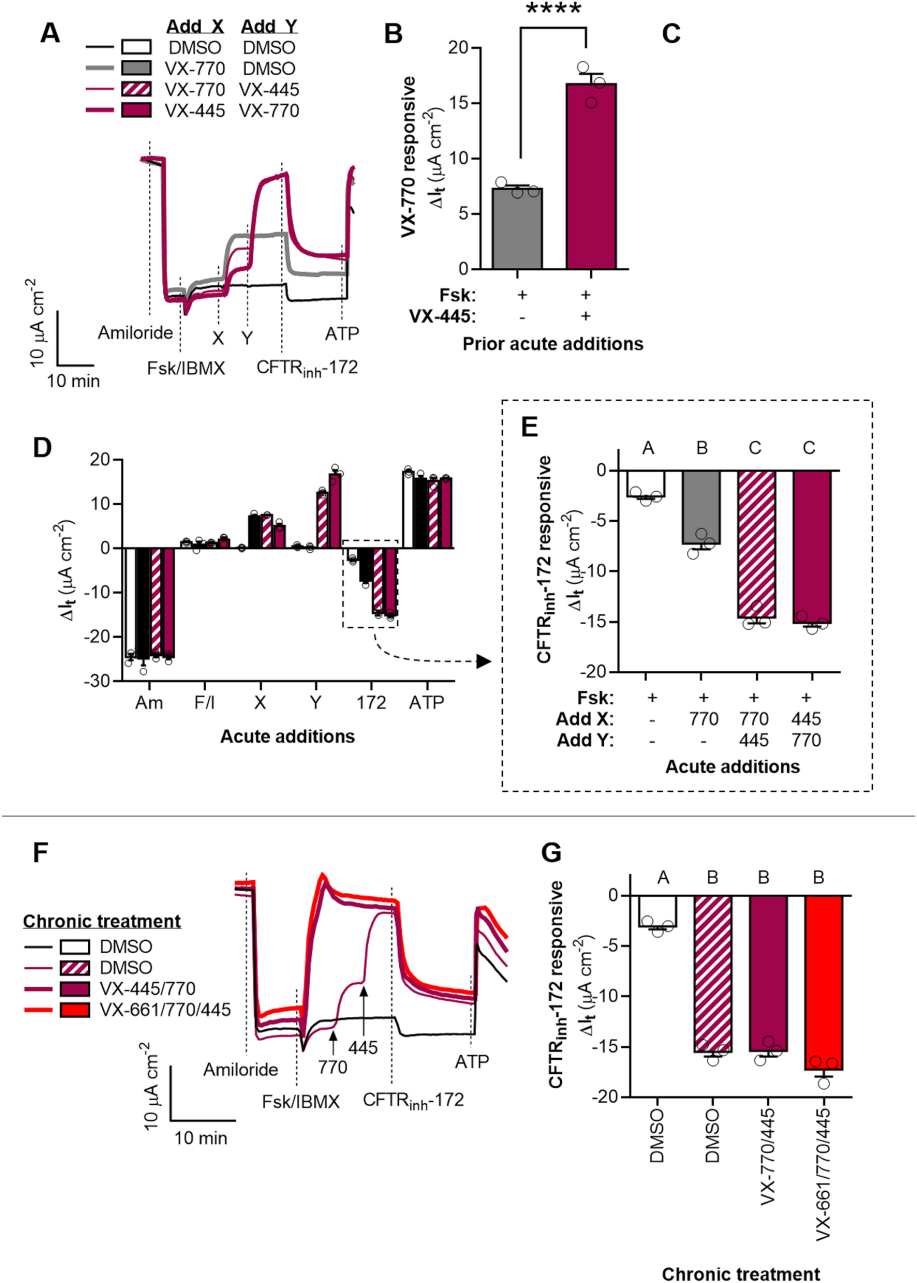
Synergism of ivacaftor (VX-770) and elexacaftor (VX-445) in potentiating G551D-CFTR in HNE cells. (**A**) Representative I_t_ recordings of G551D-HNE cells showing acute actions of VX-770 and VX-445. (**B–C**) Changes in I_t_ after acute addition of VX-770 in the absence and presence of VX-445 (**B**) and in response to the acute addition of VX-445 in the absence and presence of VX-770 (**C**). (**D–E**) Changes in I_t_ after the additions of test compounds for the experiment presented in (**A)**. G551D-CFTR mediated I_t_ is greatest after acute potentiation by both VX-770 and VX-445. (**F**) Representative I_t_ recordings of G551D-HNE treated for 24 h with DMSO, the double combination of VX-770 and VX-445, or the triple combination of VX-661, VX-770, and VX-445 (i.e., Trikafta). (**G**) CFTR_inh_-172 inhibited I_t_ for the experiment presented in (**F)**. See SI for additional experimental details and for supporting data. All data are presented as mean ± standard error. Bars with different letters (A, B, C…) are significantly different from each other (ANOVA; *P* < 0.05). Asterisks indicate specific *P* values: ****P < 0.0001.

The original Article has been corrected.

