# Supplementary material for: Elexacaftor is a CFTR potentiator and acts synergistically with ivacaftor during acute and chronic treatment
Source: Sci Rep. 2021 Oct 6;11:19810. doi: 10.1038/s41598-021-99184-1 (PMC8494914; doi:10.1038/s41598-021-99184-1)
Supplement: Supplementary file 1 — Supplementary Information. [file 41598_2021_99184_MOESM1_ESM.docx]

**SUPPLEMENTARY MATERIALS FOR**

Elexacaftor is a CFTR potentiator and acts synergistically with ivacaftor during acute and chronic treatment

Ciaran A. Shaughnessy^1^*, Pamela L. Zeitlin^1,2^, Preston E. Bratcher^1,2^

^1^Department of Pediatrics, National Jewish Health; Denver, CO.

^2^ Department of Pediatrics, University of Colorado Anschutz Medical Center; Aurora, CO

*Corresponding author. Email: ShaughnessyC@NJHealth.org.

**LIST OF SUPPLEMENTARY MATERIALS**

Supplementary Text

Supplementary Materials and Methods

Figure S1: Dose-response curve of I_t_ in non-CF HNE acutely titrated with elexacaftor (VX-445) or other established CFTR potentiators.

Figure S2: Supporting data corresponding to main text Fig. 1.

Figure S3: Supporting data corresponding to main text Fig. 2.

Figure S4: Acute and chronic actions of elexacaftor (VX-445) in WT- and F508del-FRT cell cultures.

Figure S5: Supporting data corresponding to main text Fig. 3.

Figure S6: Ivacaftor (VX-770) and elexacaftor (VX-445) potentiate R117H-CFTR expressed in FRT cells.

Figure S7: Supporting data corresponding to main text Fig. 4.

Figure S8: Chronic treatment with the combination of ivacaftor (VX-770) and elexacaftor (VX-445) increases G551D-CFTR mediated I_t_ in HNE.

Figure S9: Differences in baseline and amiloride-responsive transepithelial potential between non-CF and F508del-HNE are attenuated by CFTR modulators.

Supplementary Bibliography

Supplementary TEXT

Translatability of *in vitro* CFTR functional studies using electrophysiology

The high translatability of *in vitro* CFTR functional analyses has led to the U.S. Food and Drug Administration to expand drug labels for CF modulators to include treatment of individuals with rarer CF-causing CFTR mutations on the basis of *in vitro* studies alone, in place of further clinical testing [1–6]. Much of the translational work with CFTR modulators has centered around the development of CFTR correctors to stabilize misfolded F508del-CFTR and enhance its trafficking to the cell surface. A common experimental endpoint *in vitro* to gauge the efficacy of rescue of F508del-CFTR by CFTR correctors is the differential banding pattern of CFTR in Western blotting analyses―rescue of F508del-CFTR processing can be quantified by comparing banding of B-type (immature) to C-type (mature, past-Golgi) CFTR [7–11]. However, in the development of VX-770, which is a CFTR potentiator not a CFTR corrector, comparison of bands B and C in Western blotting was not a relevant endpoint to measure, and so these studies relied more heavily on electrophysiological investigations [12,13]. As the present study is seeking to characterize the potentiating actions of VX-445, electrophysiological investigations have played a similarly important role. Thus, it is important to further discuss the translatability of electrophysiological analysis and offer concrete examples for how our results *in vitro* may translate *in vivo*.

Nasal potential difference (NPD) testing is a tool used by clinicians to diagnose CF. In this test, a Ringer’s solution is perfused against the nasal inferior turbinate and the PD across the nasal epithelium can be measured *in vivo*. The PD at baseline, as well as the ΔPD in response to the additions of amiloride (reflective of ENaC function) and isoproterenol (reflective of CFTR function) to the Ringer’s perfusion can all be used to identify differences between non-CF and CF, as well as efficacy of CFTR modulators in increasing CFTR function [14–16]. The PD at baseline of individuals with CF (approximately -40 mV) is more negative than that of non-CF individuals (approximately -20 mV). It was shown in clinical trials that treatment with VX-770 increased PD at baseline, decreased the ΔPD response to amiloride, and increased the ΔPD response to isoproterenol [13,17]. Results from NPD testing closely correlate with results from sweat chloride and lung function analyses, which are other classically used tools in diagnosing CF [16,18].

It has been demonstrated that *in vitro* experiments using primary-derived epithelial cultures obtained from nasal brushings are suitable surrogates for predicting CFTR modulator response, and thus have a high degree of clinical relevance [19]. Analysis of PD in our HNE cultures *in vitro* supports the translatability of our results. For example, like what is observed by NPD testing *in vivo*, our CF HNE cultures *in vitro* have more negative PD at baseline and a greater ΔPD response to amiloride than non-CF HNE cultures [20] (Fig. S9*A*–*B*). Treatment of our F508del-HNE cultures with the double combination of VX-661 and VX-770 (i.e., Symdeko) or the triple combination of VX-661, VX-770, and VX-445 (I.e., Trikafta) resulted in a shift toward non-CF-like electrophysiological properties. Specifically, F508del-HNE cultures treated with the triple combination had in a less negative PD at baseline and a reduced ΔPD response to amiloride, compared to a DMSO control (Fig. S9*C–D*), reflective of what has been observed by NPD testing in individuals with CF on CFTR modulators [13,17]. Thus, our analyses of PD in our cultures *in vitro*, which are perhaps the most directly comparable elements to corresponding CFTR function analyses *in vivo*, indicate that the results from our experimental treatments with VX-770 and VX-445 in restoring channel function to G551D-CFTR are likely to translate to similar results *in vivo*.

Supplementary Materials and Methods

Cell expansion and maintenance

Human nasal epithelial cells were obtained from non-CF individuals and individuals homozygous for F508del or G551D CFTR mutations by nasal brushing as approved by the Institutional Review Board of National Jewish Health (HS-2832). These primary human airway epithelial (HNE) cells were expanded *in vitro* using an irradiated NIH 3T3 feeder layer and the Y-27632 Rho-kinase inhibitor. We seeded cells on bovine collagen-coated cell culture inserts at 2.5 x 10^5^ cells cm^-2^ submerged in Pneumacult™-EX Plus Medium (Stem Cell Technologies, Vancouver, BC). All cells were plated on 0.33 cm diameter cell culture inserts (Costar Snapwell™, Corning Inc., Kennebunk, ME) and kept at 37 °C with culture media changes 3 times per week. After 2 d, the apical solution was removed to establish an air-liquid interface (ALI), and the basolateral solution was replaced by Pneumacult™-ALI Medium (Stem Cell Technologies). We raised cells at ALI for 21–28 d at prior to electrophysiological analyses.

Fisher rate thyroid (FRT) cells expressing normal CFTR (WT-FRT), F508del-CFTR (F508del-FRT), G551D-CFTR (G551D-FRT), or R117H-CFTR (R117H-FRT) were a generous gift from Dr. Eric J. Sorscher (Emory University, Atlanta, GA). We seeded FRT cells in cell culture inserts at 1.5 x 10^4^ cells cm^-2^. The cells were maintained submerged in media for 7–10 d prior to electrophysiological analyses, with media changes every 2–3 d. The media was a modified Ham’s F-12 media (Coon’s modification) supplemented with 10% fetal bovine serum, 1% penicillin/streptomycin, and 0.2% hygromycin.

Electrophysiological analyses and drug treatment

Chronic treatment with CFTR modulators consisted of a 24 h incubation prior to electrophysiological analyses. Compound used during chronic treatment were as follows: DMSO (volume-matched), tezacaftor (VX-661; 3 µM), ivacaftor (VX-770; 100 nM), and elexacaftor (VX-445; 3 μM).

We pre-warmed (to 37 °C) cell-free inserts in an Ussing chamber apparatus (VCC MC8 and MC6; Physiologic Instruments, San Diego, CA). For all experiments, the electrode offset potential and fluid resistance were stabilized and corrected to zero with identical Ringer’s solution in the apical and basolateral baths. Ringer’s solution consisted of: 120 mM NaCl, 10 mM D-glucose, 3.3 mM KH_2_PO_4_, 0.83 mM K_2_HPO_4_, 1.2 mM MgCl_2_, 1.2 mM CaCl_2_, saturated with 95% O_2_/5% CO_2_, pH 7.4. Once electrode offset potential and fluid resistance values were zeroed, monolayers on inserts were taken directly from culture and mounted onto the Ussing chamber and allowed to stabilize under an open circuit before beginning a run under the current clamp mode.

HNE cultures are inherently polarized epithelia, mimicking the *in vivo* condition of these cells. Therefore, a basolateral-to-apical directionality of chloride transport is intrinsic to these primary-derived epithelia cultures. In contrast, FRT cells do not form polarized epithelia. Thus, the presence of a chloride gradient between the basolateral and apical baths is necessary to establish directionality of chloride transport across the FRT epithelia. Therefore, experiments using FRT cells were all performed under a chloride gradient (chloride-free in apical bath). The chloride-free Ringer’s solution consisted of: 11.5 mM Na^+^ gluconate, 5 mM Ca^2+^ gluconate, 10 mM D-glucose, 3.3 mM KH_2_PO_4_, 0.83 mM K_2_HPO_4_, 1.2 mM MgSO_4_, saturated with 95% O_2_/5% CO_2_, pH 7.4.

As depicted in figures and figure legends, the following test compounds were applied acutely in electrophysiological assays: DMSO (volume-matched); amiloride (100 μM; apical); VX-661 (see dose curve; apical and basolateral); VX-809 (see dose curve; apical and basolateral); VX-770 (see dose curve, or 1 μM; apical); VX-445 (see dose curve, or 100 nM; apical and basolateral); genistein (see dose curve; apical); forskolin (20 μM) and 3-isobutyl-1-methylxanthine (100 μM) (Fsk/IBMX or F/I; apical and basolateral); CFTR_inh_-172 (10 μM; apical); adenosine triphosphate (ATP; 100 μM; apical). In the above list, the references to sidedness of application refer to HNE cultures only; as CFTR is constitutively expressed on all plasma membrane surfaces in FRT epithelia, all compounds were applied to both surfaces of FRT cultures. Amiloride is a selective inhibitor of the epithelial sodium channel (ENaC). VX-661 and VX-809 are selective CFTR correctors. VX-770 and genistein are selective CFTR potentiators that increases the open probability of CFTR [12,21]. Fsk and IBMX together increase intracellular cAMP by activating adenylate cyclase (Fsk) and inhibiting phosphodiesterases (IBMX) and are commonly used as experimental activators of CFTR. CFTR_inh_-172 is a selective inhibitor of CFTR. ATP increases intracellular calcium and is commonly used as an indicator of calcium-activated chloride channel (CaCC) function.

In non-CF and Trikafta-treated F508del/F508del epithelia, the potentiator was added prior to Fsk/IBMX. In airway epithelia *in vivo*, a portion of the CFTR present is actively transporting chloride in the absence of exogenous forskolin. Thus, the application of a potentiator before Fsk/IBMX allows for additional functional characterization of the channel. For instance, in non-CF airway epithelia, minimal (or no) response to VX-770 is observed when Fsk/IBMX is added first [22–27].

In addition to assessing their responsiveness to test compounds, the adequacy and integrity of HNE cultures for electrophysiological analyses were determined by the presence of mucosal secretions, beating cilia, and an adequate transepithelial resistance (> 100 Ω cm^-2^). Fsk/IBMX- and CFTR_inh_-172-sensitive responses in I_t_ were considered measures of CFTR activity. All recordings were digitally acquired using Acquire and Analyze 2.3 software (Physiologic Instruments).

Supplementary Figures


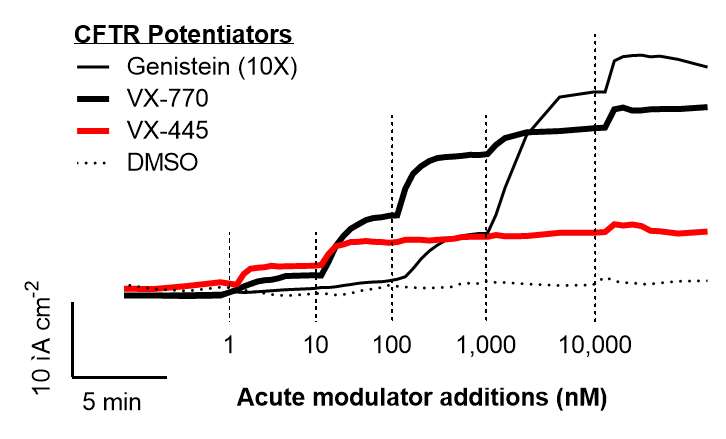


Figure S1: Dose-response curve of I_t_ in non-CF HNE acutely titrated with elexacaftor (VX-445) or other established CFTR potentiators. Acute application of VX-445 increased I_t_ across non-CF HNE in a dose-dependent manner. The maximal potentiating effect of VX-445 was less than the potentiating effects of ivacaftor (VX-770) and genistein. Note: all concentrations for genistein are 10-fold higher than indicated.


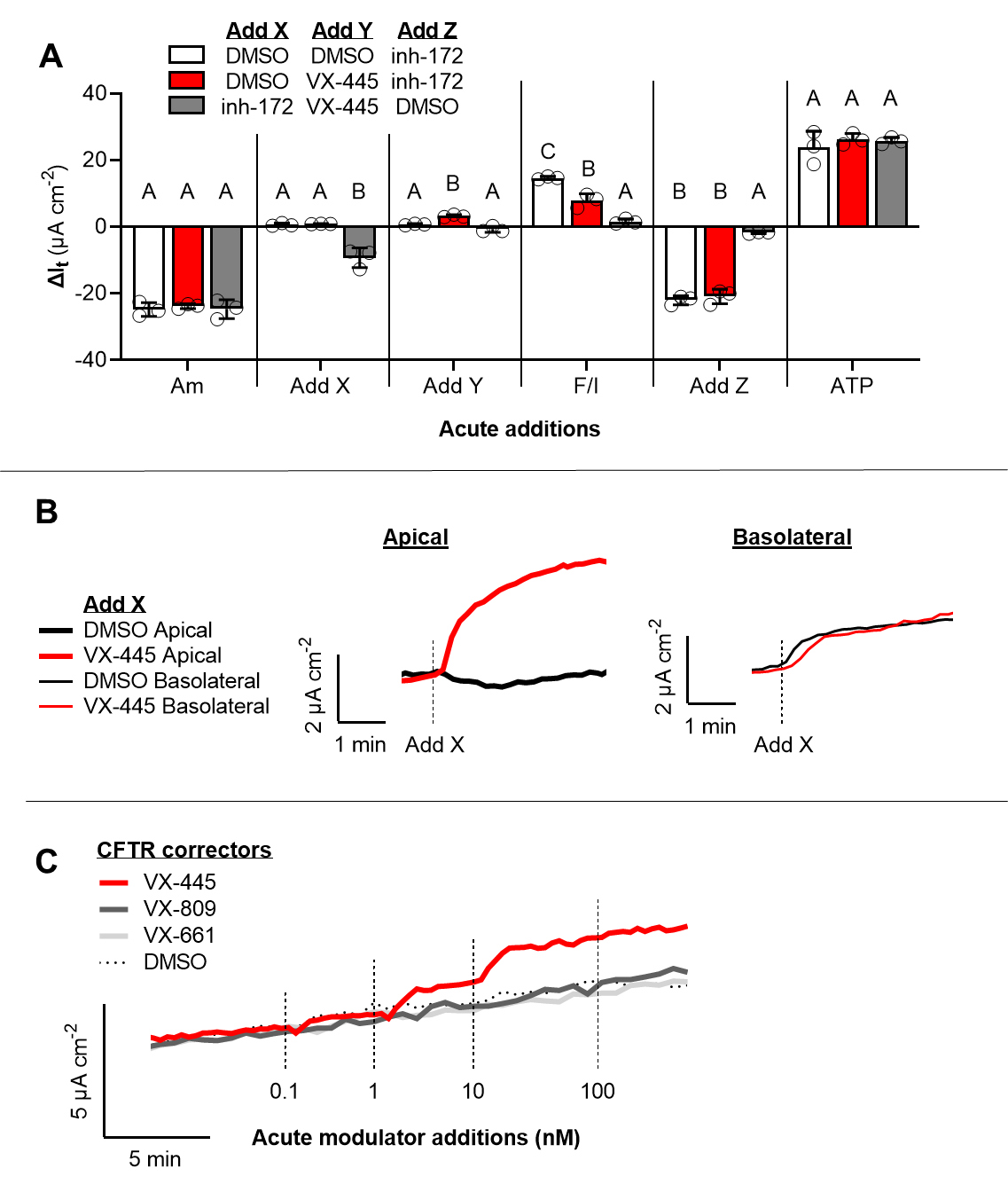


Figure S2: Supporting data corresponding to main text Fig. 1. (*A*) Details of statistical comparisons for data presented in Fig. 1*B*. (*B*) Representative I_t_ recordings corresponding to data presented in Fig. 1*E*. (*C*) Representative I_t_ recordings corresponding to data presented in Fig. 1*F*. All data is presented as mean ± standard error. Within each test compound addition, bars with different letters (A, B, C…) are significantly different (ANOVA; *P* < 0.05).


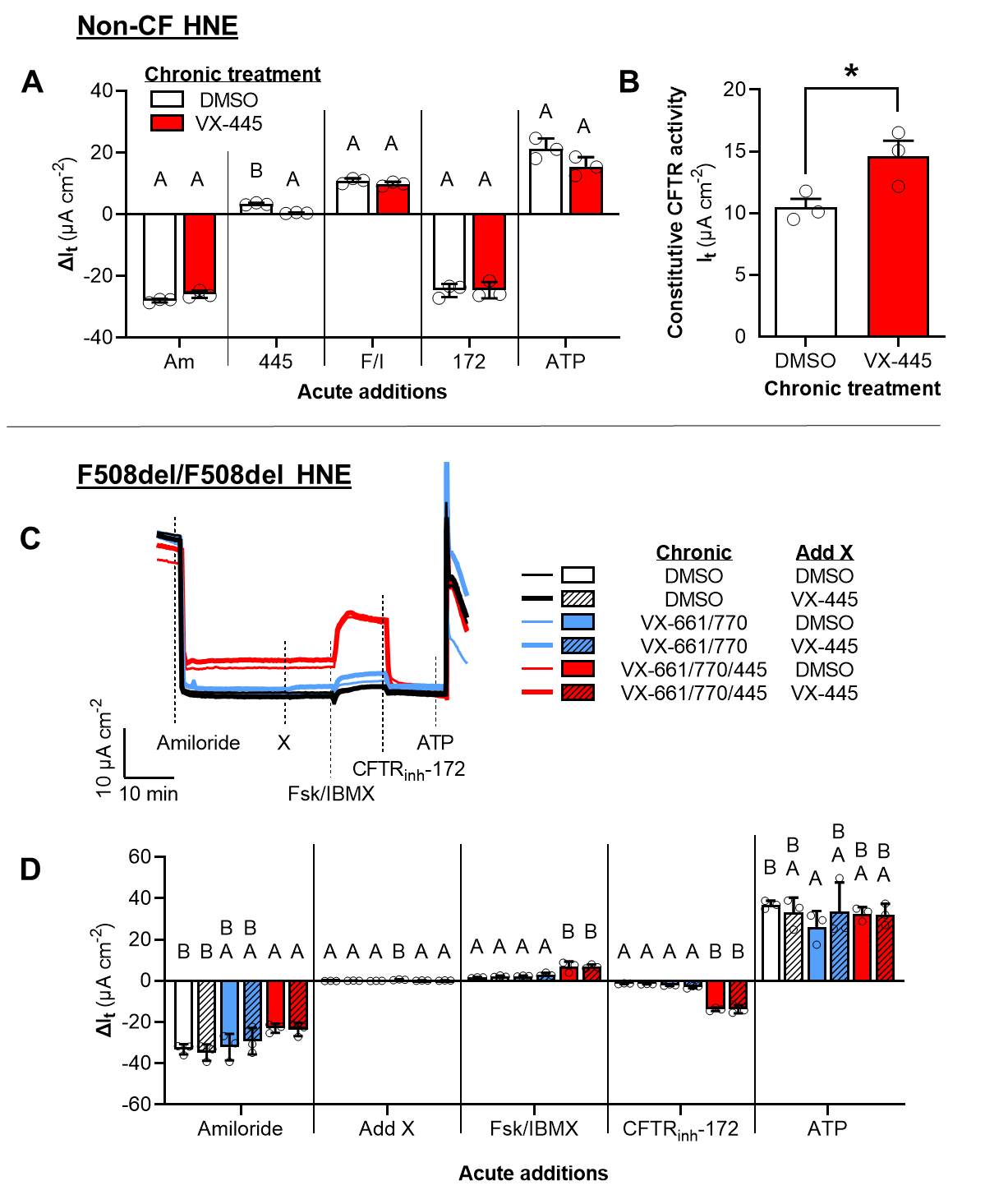


Figure S3: Supporting data corresponding to main text Fig. 2. (*A*) Details of statistical comparisons for data presented in Fig. 2*B*. (B) Constitutive CFTR activity for the experiment presented in Fig. 2A, calculated as the difference in I_t_ from values obtained after amiloride exposure to values obtained after CFTR_inh_-172 exposure. (*C*) Un-cropped I_t_ recordings corresponding to data presented in Fig. 2*D*, showing the amiloride and ATP responses. (*D*) Details of statistical comparisons for data presented in Panel *C*. All data are presented as mean ± standard error. Within each test compound addition, bars with different letters (A, B, C…) are significantly different (ANOVA; *P* < 0.05).


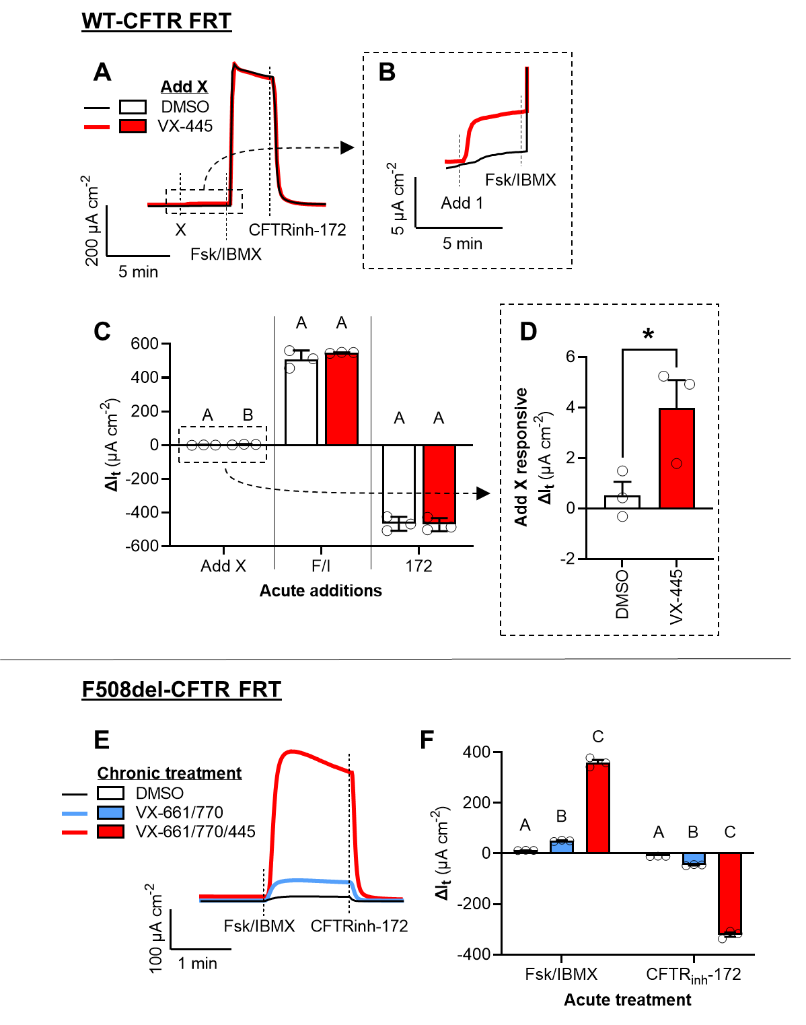


Figure S4: Acute and chronic actions of elexacaftor (VX-445) in WT- and F508del-FRT cell cultures. (A–B) Representative I_t_ recordings of FRT cells expressing normal CFTR after the acute additions of DMSO, VX-445, Fsk/IBMX, and/or CFTR_inh_-172. (C–D) Changes in I_t_ after the additions of test compounds for the experiment presented in Panel *A*. (*D*) Representative I_t_ recordings of FRT cells expressing F508del-CFTR treated for 24 h with DMSO, the double combination of tezacaftor (VX-661) and ivacaftor (VX-770) (i.e., Symdeko) or the triple combination of VX-661, VX-770, and VX-445 (i.e., Trikafta). (*F*) Changes in I_t_ after the additions of test compounds for the experiment presented in Panel *E*. All data are presented as mean ± standard error. Within each test compound addition, bars with different letters (A, B, C…) are significantly different (ANOVA; *P* < 0.05).


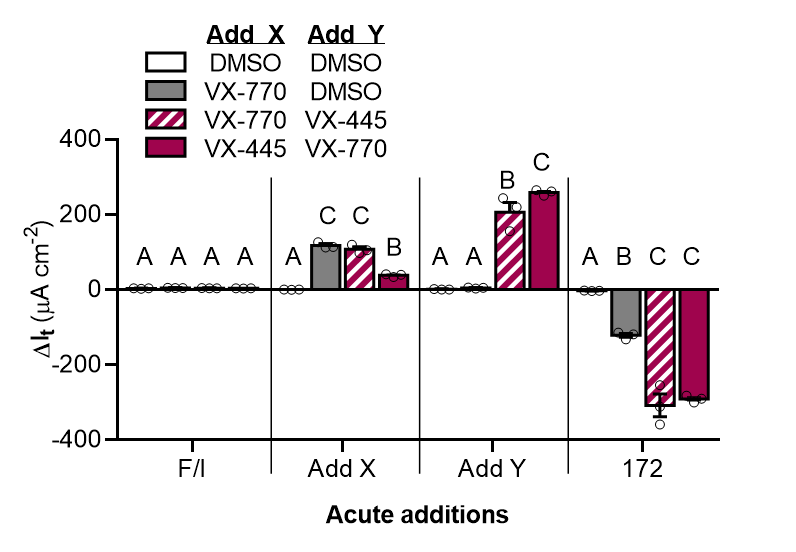


Figure S5: Supporting data corresponding to main text Fig. 3. Details of statistical comparisons for data presented in Fig. 3*D*. All data are presented as mean ± standard error. Within each test compound addition, bars with different letters (A, B, C…) are significantly different (ANOVA; *P* < 0.05).


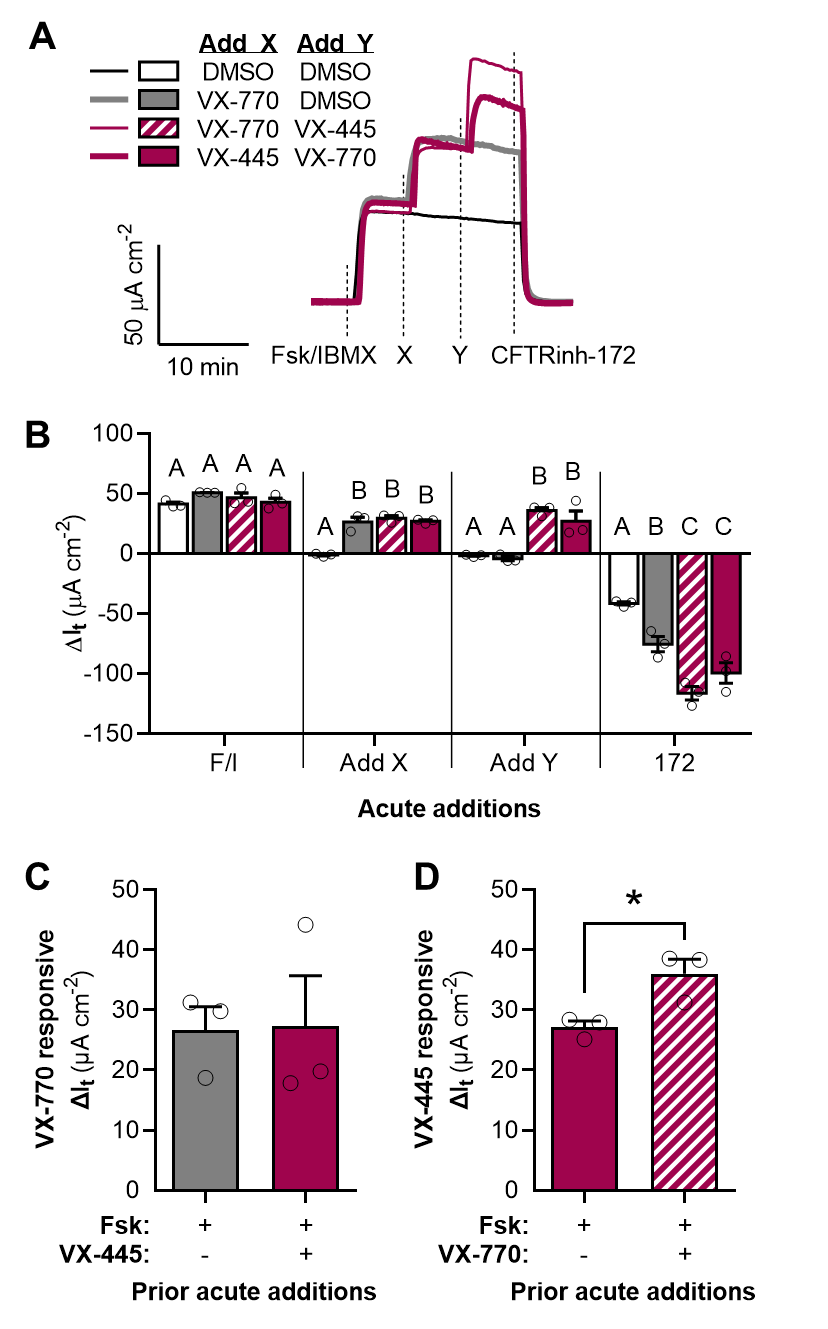


Figure S6: Ivacaftor (VX-770) and elexacaftor (VX-445) potentiate R117H-CFTR expressed in FRT cells. (*A*) Representative I_t_ recordings of R117H-FRT cells showing acute actions of VX-770 and VX-445. (*B*) Changes in I_t_ after the additions of test compounds for the experiment presented in Panel *A*. R117H-CFTR mediated I_t_ is greatest after acute potentiation by both VX-770 and VX-445. (C–D) Changes in I_t_ after acute addition of VX-770 in the absence and presence of VX-445 (C) and in response to the acute addition of VX-445 in the absence and presence of VX-770 (D). All data are presented as mean ± standard error. Within each test compound addition, bars with different letters (A, B, C…) are significantly different (ANOVA; *P* < 0.05).


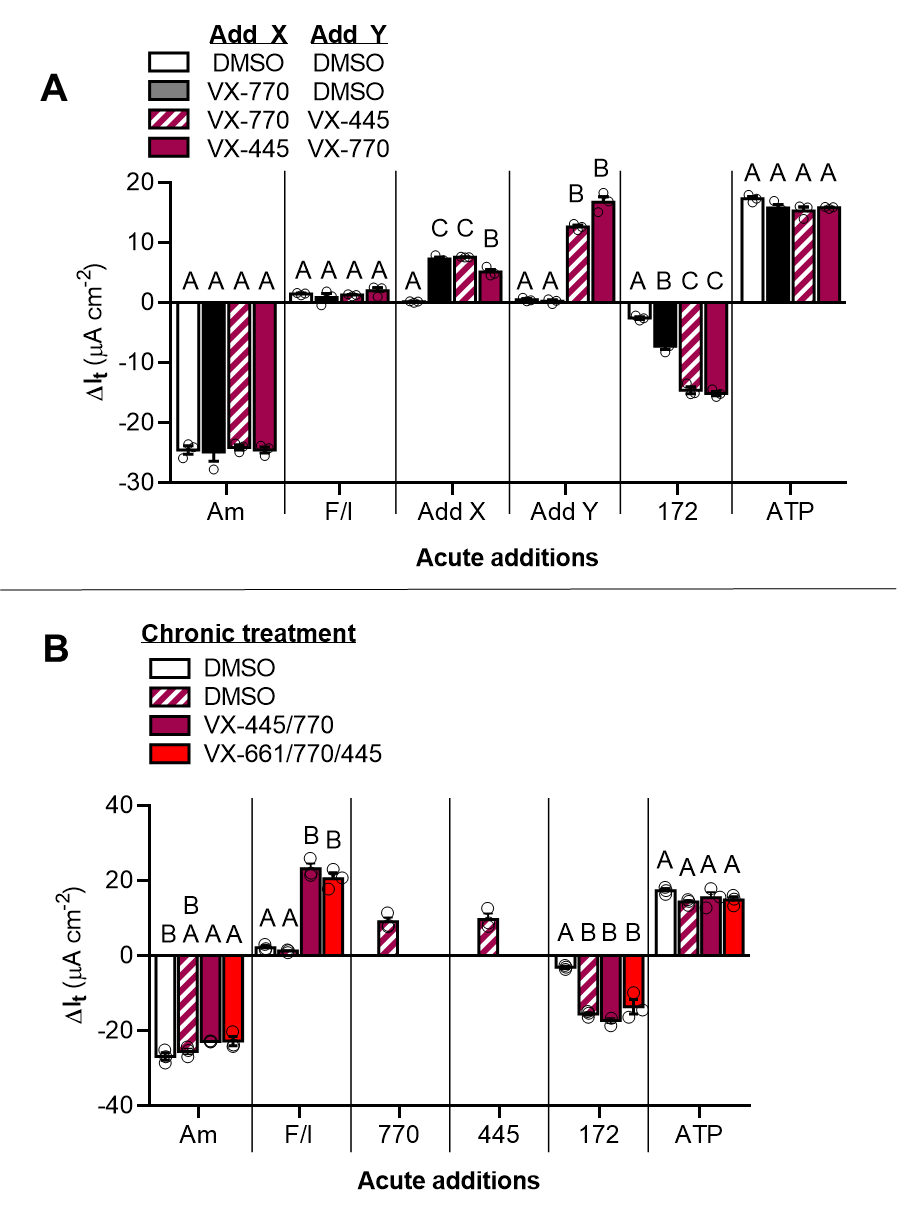


Figure S7: Supporting data corresponding to main text Fig. 4. (*A*) Details of statistical comparisons for data presented in Fig. 4*D*. (*B*) Changes in I_t_ after the additions of test compounds for the experiment presented in Fig 4*F*. All data are presented as mean ± standard error. Within each test compound addition, bars with different letters (A, B, C…) are significantly different (ANOVA; *P* < 0.05).


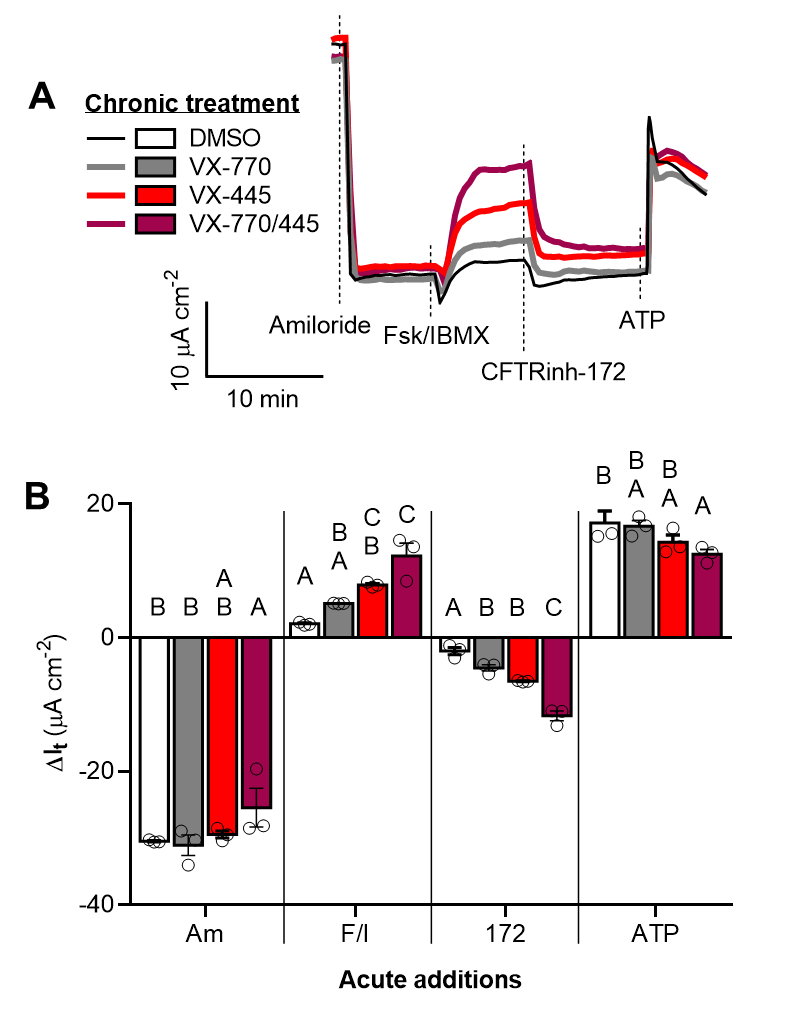


Figure S8: Chronic treatment with the combination of ivacaftor (VX-770) and elexacaftor (VX-445) increases G551D-CFTR mediated I_t_ in HNE. (*A*) Representative I_t_ recordings of G551D-HNE treated for 24 h with DMSO, VX-770, and/or VX-445. (*B*) Changes in I_t_ after the addition of CFTR_inh_-172 in the experiment presented in Panel *A*. G551D-CFTR mediated I_t_ is greatest after chronic treatment by both VX-770 and VX-445. All data are presented as mean ± standard error. Within each test compound addition, bars with different letters (A, B, C…) are significantly different (ANOVA; *P* < 0.05).


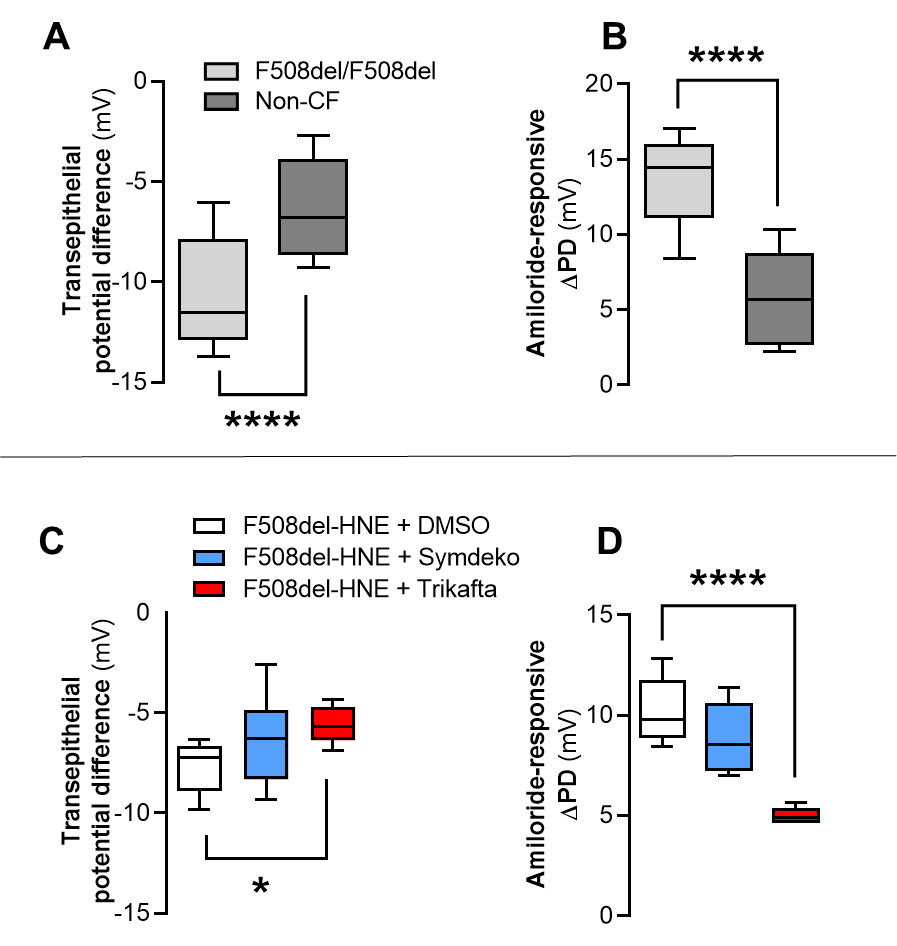


Figure S9: Differences in baseline and amiloride-responsive transepithelial potential between non-CF and F508del-HNE are attenuated by CFTR modulators. (*A–B*) Comparisons of baseline transepithelial potential difference (PD) (*A*) and ΔPD in response to amiloride (*B*) between non-CF HNE and F508del-HNE cultures. Original data for Panels *A* and *B* is presented in [20]. (*C–D*) Comparisons baseline PD (*C*) and ΔPD in response to amiloride (D) in F508del-HNE treated for 24 h with DMSO, the double combination of tezacaftor (VX-661) and ivacaftor (VX-770) (i.e., Symdeko) or the triple combination of VX-661, VX-770, and VX-445 (i.e., Trikafta). All data are presented as mean (line), quartiles (box), and minimum and maximum values (bars). Asterisks indicate differences (ANOVA with Tukey’s *post hoc* comparisons): * *P* < 0.05; **** *P* < 0.0001.

**SUPPLEMENTARY BIBLIOGRAPHY**

1 Kingwell K. FDA OKs first in vitro route to expanded approval. Nat Rev Drug Discov. 2017;16(9):591–2.

2 Durmowicz T, Pacanowski M. Novel Approach Allows Expansion of Indication for Cystic Fibrosis Drug [Internet]. FDA News Events. 2017Available from: https://www.fda.gov/Drugs/NewsEvents/ucm559051.htm

3 Durmowicz AG, Lim R, Rogers H, Rosebraugh CJ, Chowdhury BA. The U.S. food and drug administration’s experience with ivacaftor in cystic fibrosis: Establishing efficacy using in vitro data in lieu of a clinical trial. Ann Am Thorac Soc. 2018;15(1):1–2.

4 Vertex Pharmaceuticals Incorporated. Prescribing Information: KALYDECO® (ivacaftor) tablets, for oral use. 2020Available from: https://pi.vrtx.com/files/uspi_ivacaftor.pdf

5 Vertex Pharmaceuticals Incorporated. Prescribing Information: SYMDEKO® (tezacaftor/ivacaftor) tablets; (ivacaftor) tablets, for oral use. 2019Available from: https://pi.vrtx.com/files/uspi_tezacaftor_ivacaftor.pdf

6 Vertex Pharmaceuticals Incorporated. Prescribing Information: TRIKAFTA® (elexacaftor, tezacaftor, and ivacaftor tablets; ivacaftor tablets), co-packaged for oral use. 2021Available from: https://pi.vrtx.com/files/uspi_elexacaftor_tezacaftor_ivacaftor.pdf

7 Van Goor F, Hadida S, Grootenhuis PDJ, Burton B, Stack JH, Straley KS, et al. Correction of the F508del-CFTR protein processing defect in vitro by the investigational drug VX-809. Proc Natl Acad Sci U S A. 2011;108(46):18843–8.

8 Wainwright CE, Elborn JS, Ramsey BW, Marigowda G, Huang X, Cipolli M, et al. Lumacaftor-ivacaftor in patients with cystic fibrosis homozygous for phe508del CFTR. N Engl J Med. 2015;373(3):220–31.

9 Taylor-Cousar JL, Munck A, McKone EF, Van Der Ent CK, Moeller A, Simard C, et al. Tezacaftor–ivacaftor in patients with cystic fibrosis homozygous for Phe508del. N Engl J Med. 2017;377(21):2013–23.

10 Donaldson SH, Pilewski JM, Griese M, Cooke J, Viswanathan L, Tullis E, et al. Tezacaftor/ivacaftor in subjects with cystic fibrosis and F508del/F508del-CFTR or F508del/G551D-CFTR. Am J Respir Crit Care Med. 2018;197(2):214–24.

11 Keating D, Marigowda G, Burr L, Daines C, Mall MA, McKone EF, et al. VX-445-tezacaftor-ivacaftor in patients with cystic fibrosis and one or two Phe508del alleles. N Engl J Med. 2018;379(17):1612–20.

12 Van Goor F, Hadida S, Grootenhuis PDJ, Burton B, Cao D, Neuberger T, et al. Rescue of CF airway epithelial cell function in vitro by a CFTR potentiator, VX-770. Proc Natl Acad Sci U S A. 2009;106(44):18825–30.

13 Accurso FJ, Rowe SM, Clancy JP, Boyle MP, Dunitz JM, Durie PR, et al. Effect of VX-770 in Persons with Cystic Fibrosis and the G551D- CFTR Mutation. N Engl J Med. 2010;363(21):1991–2003.

14 Rowe SM, Clancy JP, Wilschanski M. Nasal Potential Difference Measurements to Assess CFTR Ion Channel Activity. In: Amaral MD, Kunzelmann K, editors. Cystic Fibrosis: Diagnosis and Protocols, Volume I: Approaches to Study and Correct CFTR Defects. Totowa, NJ: Humana Press; 2011; pp 69–86.

15 Alton EWFW, Currie D, Logan-Sinclair R, Warner JO, Hodson ME, Geddes DM. Nasal potential difference: A clinical diagnostic test for cystic fibrosis. Eur Respir J. 1990;3(8):922–6.

16 Ho LP, Samways JM, Porteous DJ, Dorin JR, Carothers A, Greening AP, et al. Correlation between nasal potential difference measurements, genotype and clinical condition in patients with cystic fibrosis. Eur Respir J. 1997;10(9):2018–22.

17 Rowe SM, Liu B, Hill A, Hathorne H, Cohen M, Beamer JR, et al. Optimizing Nasal Potential Difference Analysis for CFTR Modulator Development: Assessment of Ivacaftor in CF Subjects with the G551D-CFTR Mutation. PLoS One. 2013;8(7). DOI: 10.1371/journal.pone.0066955

18 Accurso FJ, Van Goor F, Zha J, Stone AJ, Dong Q, Ordonez CL, et al. Sweat chloride as a biomarker of CFTR activity: Proof of concept and ivacaftor clinical trial data. J Cyst Fibros. 2014;13(2):139–47.

19 Pranke I, Hatton A, Masson A, Flament T, Le Bourgeois M, Chedevergne F, et al. Might brushed nasal cells be a surrogate for CFTR modulator clinical response? Am J Respir Crit Care Med. 2019;199(1):123–6.

20 Yadav S, Shaughnessy CA, Zeitlin PL, Bratcher PE. Downregulation of epithelial sodium channel (ENaC) activity in human airway epithelia after low temperature incubation. BMJ Open Respir Res. 2021;8:e000861.

21 Illek B, Zhang L, Lewis NC, Moss RB, Dong J, Fischer H. Defective function of the cystic fibrosis-causing missense mutation G551D is recoved by genistein. Am J Physiol - Cell Physiol. 1999;46:833–9.

22 Bratcher PE, Yadav S, Shaughnessy CA, Thornell IM, Zeitlin PL. Effect of apical chloride concentration on the measurement of responses to CFTR modulation in airway epithelia cultured from nasal brushings. Physiol Rep. 2020;8:e14603.

23 Eckford PDW, Li C, Ramjeesingh M, Bear CE. Cystic fibrosis transmembrane conductance regulator (CFTR) potentiator VX-770 (ivacaftor) opens the defective channel gate of mutant CFTR in a phosphorylationdependent but ATP-independent manner. J Biol Chem. 2012;287(44):36639–49.

24 Cholon DM, Quinney NL, Fulcher ML, Esther CR, Das J, Dokholyan N V., et al. Potentiator ivacaftor abrogates pharmacological correction of ΔF508 CFTR in cystic fibrosis. Sci Transl Med. 2014;6(246):1–12.

25 Veit G, Avramescu RG, Perdomo D, Phuan PW, Bagdany M, Apaja PM, et al. Some gating potentiators, including VX-770, diminish ΔF508-CFTR functional expression. Sci Transl Med. 2014;6(246):1–14.

26 Cui G, Stauffer BB, Imhoff BR, Rab A, Hong JS, Sorscher EJ, et al. VX-770-mediated potentiation of numerous human CFTR disease mutants is influenced by phosphorylation level. Sci Rep. 2019;9(1):1–17.

27 Pyle LC, Ehrhardt A, Mitchell LH, Fan L, Ren A, Naren AP, et al. Regulatory domain phosphorylation to distinguish the mechanistic basis underlying acute CFTR modulators. Am J Physiol - Lung Cell Mol Physiol. 2011;301(4):587–97.
